# Net profit flow per country from 1980 to 2009: The long-term effects of foreign direct investment

**DOI:** 10.1371/journal.pone.0179244

**Published:** 2017-06-27

**Authors:** Dirk H. M. Akkermans

**Affiliations:** Department of Global Economics and Management, Faculty of Economics and Business, University of Groningen, Groningen, the Netherlands; Universidad Veracruzana, MEXICO

## Abstract

**Aim of the paper:**

The paper aims at describing and explaining net profit flows per country for the period 1980–2009. Net profit flows result from Foreign Direct Investment (FDI) stock and profit repatriation: inward stock creating a profit outflow and outward FDI stock a profit inflow. Profit flows, especially ‘normal’ ones are not commonly researched.

**Theoretical background:**

According to world-system theory, countries are part of a system characterised by a core, semi-periphery and periphery, as shown by network analyses of trade relations. Network analyses based on ownership relations of TransNational Corporations (TNCs) show that the top 50 firms that control about 40% of the world economy are almost exclusively located in core countries. So, we may expect a hierarchy in net profit flows with core countries on top and the periphery at the bottom. FDI outflows from the core countries especially rose in the 1990s, so we may expect that the difference has grown in time.

**Data and results:**

A dataset on 'net profit flow' per country is developed. There are diverging developments in net profit flows since the 1980s, as expected: ever more positive for core countries, negative and ever lower for semi-peripheral and peripheral countries, in particular from the 1990s onwards. A fixed effects quantile regression using publicly available data confirms the prediction that peripheral countries share a unique characteristic: their outward investments do not have a positive influence on net profit flow as is the case with semi-peripheral and core countries. The most probable explanation is that peripheral outward investments are indirectly owned by firms located in core and semi-peripheral countries, so all peripheral profit inflows end up in those countries.

## Introduction

Literature shows two opposite views on the effects of Foreign Direct Investment (FDI) on the economies of host countries. FDI would constitute an inflow of capital, (management) knowledge and technology, and hence would boost economic growth of developing countries in particular as these countries would suffer from shortages in these areas [1,2]. The empirical record has supported that notion; however, also negative relationships have been found for developing countries (e.g. [3–9], pointing at a detrimental effect of FDI. Repatriation of profits–also called ‘drain of wealth’–is among the possible harmful effects of FDI.

The present paper focuses on analysing level and development of net profit in- or outflow of countries–‘drain of wealth’–in 1980–2009. Profit flows are hardly investigated; illicit–illegal [10]–capital flows related to tax havens have received most of the attention. We shall investigate the ‘normal’ profit flows, meaning the flows that are officially registered as such and shall look into a number of factors that might explain them. Second, the paper investigates a large part of the period of neoliberalism, 1980–2009 [11]. This period witnessed a rise in FDI from 1990 onwards, in particular from developed countries, and changes in the institutional setup of countries such as liberalisation which might have affected changes in net profit flows between countries. The third goal is to give the most general picture possible by aiming at maximum coverage in number of countries.

### Drain of wealth – the long-term effects of FDI

The literature on FDI generally states that it might foster economic growth because of two main reasons: first, FDI means capital import, and second, it might entail a transfer of management skills, technology and knowledge to the host economy, e.g. [12]. As FDI normally originates from developed countries (outward FDI from developing countries is a recent phenomenon [13]), it has come to be viewed in research and policy as vital for development of poor countries in particular.

FDI plays a crucial role in Dunning’s Investment Development Path (IDP; [12,14] which states that “countries tend to go through five phases (from ‘least developed’ to ‘developed’), in which the propensity of being a net recipient to ultimately becoming a net source of FDI evolves (..).” ([13], p. 143). It would create a ‘process of structural upgrading driven by inward and outward FDI’, culminating in ‘growing national competitiveness’ ([13], p. 144). Hence, “(…) countries may use both inward and outward FDI to upgrade the competitiveness of their indigenous resources and capabilities, thereby promoting dynamic comparative advantage.” ([13], p. 147). In the fifth stage, the now developed country reaches equilibrium between inward and outward stock.

The IDP notion–and FDI literature–ignore two important aspects: first, most attention goes to what from the viewpoint of self-interested investors are primarily unintended consequences: economic growth of the host country and technology transfer. Profits constitute the goal of capitalist production, “The main objective of all foreign investment is to make profits and to repatriate those profits to the home state.” ([15], p. 206). And profit maximisation and repatriation do not automatically match with economic growth and/or technology transfer in the host country. We shall discuss capital import (a) and knowledge and technology transfer (b) more extensively.

(a) When a foreign firm invests in a host country and then reinvests the profits there [16,17] a multiplier effect might arise [18,19]. However, when profits are exported or repatriated instead of reinvested–repatriation might start five years after initial investment [20]–the country loses capital for accumulation and investment, which, if not compensated for by new FDI or domestic investment, will result in a negative investment multiplier [5,21]. TNCs then act as what Açemoglu and Robinson ([22], p. 70) call 'extractive institutions', 'designed to extract income and wealth'–although the term is interpreted here as an international process of wealth distribution and not a national one, as Açemoglu and Robinson intended.

Research shows that the relation between FDI and economic growth is rather tenuous. Bornschier and Chase-Dunn distinguished between FDI inward flows–new investments creating new production capacity in a country–and FDI inward stocks–productive capacity already present. They showed that new investments (FDI inward flows) influence economic growth in the host country positively, while FDI inward stocks exerted a negative influence ([5], pp. 94–95)–corroborating the idea of TNCs as extractive institutions. Blonigen and Wang [4] later found the same negative effect of FDI inward stock.

Later research amended the findings regarding FDI inward flows as the privatisations in many countries changed the composition of FDI [23]. The positive effect of FDI inflows depends on whether they constitute new investments; when it concerns the purchase of already existing productive capacity like mergers and acquisitions–and land purchases [24,25]–economic growth is not positively affected (e.g. [23,26–28]).

The link between FDI inflows and economic growth in the host country was further qualified because country-specific conditions appeared to play an important role: the relation is only positive when the financial system is well-developed [29,30], or the level of education is sufficiently high [31,32]. Other institutions that have been researched are for instance the presence of corruption and bureaucratic quality [33].

(b) The difference in technology and productivity between developed and developing countries is well-known ([13], p. 152; [34,35]); Chang ([36], p. 102) states that the productivity gap between the poorest and the richest countries has grown substantially since the 19^th^ century from 2–4 to 1 to 50–60 to 1 in 1999. Since 1980 developed countries have outsourced large parts of their manufacturing sector to low-wage developing countries: their deindustrialization meant industrialization of in particular Asian countries [37,38]. Becoming part of Global Value Chains (GVC) might enable learning [39–42], in particular when people are well educated [31] ([29,43–45]). ‘Spillover’ through horizontal or vertically upward/downward relations became a major research theme as it could provide opportunities for ‘upgrading’ [46]. Given, however, that knowledge and technology are strong competitive advantages, developed-country TNCs will minimise transfer as it would undermine their profitability; knowledge protection is standard policy [36,47–49]. Developed countries and TNCs press for protection, in particular through the Trade-Related Property Rights Agreement (TRIPS). Although article 66.2 of the agreement states that developed countries should stimulate firms and other institutions to transfer technology [50], the article is factually non-mandatory. The situation is succinctly summarised by Bashir: “(…) industrialized countries of the world hold 97% of all patents {in 2000 –the author}. However, 90 per cent of all technology and product patents are owned by multinational enterprises. On these bases, the developed economies argue that in most of the cases it is not possible for them to transfer technologies.” ([51], p. 14; see also [52,53]).

The second aspect that is ignored is the presence of power relations in the international economy. Countries and TNCs are part of an international system which is characterised by vested interests and power differentials which could–and do–create barriers to development. Theories that address the structure of this international system, e.g. world-system theory, distinguish between ‘core’ and ‘periphery’ [54,55]. The group of core countries is supposedly well connected to each other and to the periphery, while countries in the periphery are hypothesized to be isolated from each other and only connected to the core [56–58]. Power resides in the core, which exploits the periphery, so profits flow from the periphery to the core. The same applies to TNCs.

Network analyses based on trade relations in goods were used to analyse the relations between countries–see for trade dependencies between different groups of countries: S3 Appendix, in percentages. Shareholder ownership between TNCs was used as indicator of influence and control; the TNC network also provides an indication of the direction of profit flows as shareholders are entitled to dividend payments. In both cases a core-periphery structure became visible. We may expect that there is overlap between the two networks as there is a substantial amount of firm-internal trade [59,60]. Table 1 combines the results of these network analyses [61–64]:

**Table 1:** Centrality and control in the world economy.

World-system groups (*)	Nr. of top-50 controlling firms (**)
Core	47
Semi-periphery	3
Periphery	-
Countries	
United States	24
Great Britain	8
China	1
Other	17
Industry	
Financial	45
Other	5

(*) Source: Lloyd et al., 2009. World-system positions based on the year 2001. Core equals group 1, semi-periphery groups 2 and 3. See also Clark & Beckfield, 2009.

(**) Source: Vitali et al., 2011. These 50 firms exert together almost 40% of global control. The community analysis Vitali & Battiston, 2014 confirms the link between geography and control.

There is a strong match between core countries (well-developed countries like USA, UK, Netherlands, France etc.) on the one hand and central TNCs on the other: 47 of the 50 are located in core countries, and 24 in the top core country, the USA. Hence, core countries and core-based TNCs will thrive (see for the US in particular [65,66]); other countries and firms will be on the losing side.

Research shows that core governments and TNC managers influence each other and that a close match between TNC interests and government policies exists (for the US: [67–70]; for Europe [71–76]). And as development of other countries and firms might endanger the position of those of the core, we may expect that the latter will pursue a policy of ‘kicking away the ladder’ [36], sometimes in the form of full-fledged empire-oriented programmes ([77,78]; Project for a New American Century [79]).

During 1980–2009, the period of neoliberalism, FDI originating from core/developed countries grew strongly. Stimulated by core FDI flows the period showed a clear push of core governments and TNCs [80]–and IMF and World Bank [81–83]–to introduce institutional changes that served profit maximisation: stronger protection of property rights, liberalisation and deregulation and privatization, often referred to as the Washington Consensus [84,85]. Barriers to capital mobility were generally lowered, making FDI and profit repatriation easier. Revision of tax rates and other FDI-friendly measures aimed at boosting profits (e.g. [86–88]). Strengthening property rights, in particular intellectual property rights (TRIPS provided more protection for investors and specifically stronger protection for the competitive advantage of core firms, knowledge.

Two developments in this period contributed to the position of core countries and firms: first, concentration in global industries with leading firms located in the core grew [89,90] which created even more opportunities for extraction of income and wealth. Next, many African and South-American countries suffered deindustrialization, damaging prospects for development [37,38,91] as deindustrialisation makes ‘upgrading’ and technology transfer virtually impossible (Chinese imports might be an explanation for deindustrialization, for a case-study see [92]).

As this brief and incomplete overview shows, many elements have been researched regarding the role and effect of FDI in particular in developing countries. However, research into profit flows leaving (developing) countries received scarce attention, although there are exceptions [20,93].Given this background, more insight into profit flows would add to the picture that is already present.

Several hypotheses can be formulated. Each country will experience both inflow and outflow of profits as in principle any country can act as host country (inward) but also as home country (outward) of foreign investments, the net position over a number of years indicates whether a country gains from FDI or not. Given the net FDI stock position, we may expect a hierarchy in the level of net profit flow.

Hypothesis 1: The level of net profit flow will be highest for the core countries, and lowest for the peripheral countries.

The developments in FDI flows from 1990 onwards suggest that.

Hypothesis 2: Net profit outflow from peripheral and semi-peripheral countries has grown in the last two decades.

The recently growing amount of outward FDI from developing countries could create a stronger profit inflow, mitigating the growing outflow. But besides the lack of outward FDI stock that influences the balance of inward and outward profit flows for developing countries negatively it is also possible that peripheral countries are drained by their own firms–outward FDI stock creates a negative profit flow, contrary to outward FDI stock of semi-peripheral and core countries. After all, Blonigen and Wang [4] showed using interaction effects that the role of FDI stock differed between developed and developing countries: positive for developed, negative for developing countries. Possible reasons are for instance that tax havens are concentrated in core countries, while war and internal turmoil is concentrated in countries with low GDP per capita [94], creating reasons to keep the money outside the country. Summarising:

Hypothesis 3: outward FDI stock does not–or: to a lesser extent–contribute to a net profit inflow when it concerns a peripheral country.

Net profit outflow for peripheral countries, then, might be caused by on the one hand a lack of outward FDI stock and its use as capital-exporting device on the other.

## Materials and methods

### Construction of the indicator 'Net Profit Flow'

The indicator 'net profit flows' developed here is based on money flows as officially registered in the National Accounts [16]. In their 2002 study Bertocchi and Canova investigated the ‘drain of wealth’ as a consequence of colonialism and defined 'drain' as 'repatriated profits, royalties and direct exploitation activities' ([93], pp. 1852-1853/1857). They used the GNP/GDP ratio in 1960 as indicator. However, the difference between GNI and GDP contains not only profits, but also income categories as wages [16]. Therefore, we have to correct the difference GNI–GDP for net international wage flows. We shall do this by subtracting the net international wage flows from the GNI-GDP difference, using the series on ‘remittances received and paid’ to correct for wage flows. The formula used to calculate net profit flow is as follows (Formula 1):

$$Net\;Profit\;Flow_{it}=GNI_{it}-GDP_{it}-\left(Rreceived_{it}-Rpaid_{it}\right)$$ Formula 1

*where*: *i = country i*
t = year t
R = remittances
*Remittances received and paid are from and to 'the rest of the world'*.

A plus indicates a net inflow, a minus a net outflow. A real-life example from the data (all amounts in current dollars and rounded): in the year 2009 Honduras had a GDP of 14.3 billion (bn), its GNP was 13.8 bn. The GDP-GNP difference is -500 mln. Honduras received wage remittances in the height of 2.52 bln, and paid an amount of 11.6 mln. Hence, net remittances were 2.5084 bn. Consequently, net profit flow equals -3.0084 bn. which is about 21% of GDP.

Net Profit Flow (NPF) partly overestimates profit flows as it also contains all interest payments on debt. However, FDI are partly done in the form of loans that have to be repaid. These intercompany debt payments are considered to be part of Direct Investment Income (DII), the official indicator of FDI-based income (“the return on equity and debt investment”; [95], p. 74; [96], p. 40). Moreover, intercompany debt payments are often profit flows in disguise via Special Purpose Entities (SPEs) in tax havens [97–102]. So, some debt payments belong to profit flows, but not all debt payments. Hence, one could argue that DII as indicator is more precise than the one developed above because DII contains only those intercompany debt payments that stem from FDI.

There are two reasons to stick to the indicator developed. Outside the scope of intercompany debt are debt and interest payments collected by Export Credit Agencies [103–106] that countries use to pay for imports of goods. According to Eurodad these debts are substantial: almost 80 percent of debts owed by developing countries to four European stem from export credits ([107], p. 3; [108,109]). Also, a part of government debt is payment for large (infrastructural) projects contracted out to TNCs such as Halliburton and Bechtel [110–112]. All these categories contain a profit element as well. However, there is overestimation here.

As said earlier, this paper does not look at ‘illicit’ flows. The estimates of illicit profit flows that are available only concern semi-peripheral and peripheral countries. There might be some overlap between NPF and illicit profit flows because of interest payments, but it depends on the methodology used for estimating illicit capital flows [113,114].

Second, the present paper aims at maximum coverage in number of countries. A minimum of 10 observations per country for the period 1980–2009 was defined as threshold for the 'structural net position' in terms of net profit. That yields effectively 101 countries for the variable net profit flows and 66 for Direct Investment Income (DII), and 47 vs. 38 when ‘financial crises varieties’ are added to the analysis (model 2)–the latter number even too low for a statistical test of the model. We shall use DII as robustness check–see S1 Appendix.

One last remark: NPF also underestimates profit flows because profits disguised as production costs (management fees, costs of intangibles such as brand names [115]) are registered as ‘export of services’ and hence are not counted as profits–they are also absent in DII.

### Controls

The controls can be grouped into trade-related factors, financial factors, and institutional and political factors.

Trade-related factors are openness, foreign investment concentration and level of rents on total natural resources. Having an open economy–a high level of imports plus exports–will be accompanied by negative net profit flow. A large part of international trade is realised by TNCs that will repatriate profits to the core countries, which means that for the majority of countries openness is connected to loss of profits. Kentor and Boswell ([7], p. 310) showed that foreign investment concentration had a significant and long-term influence on economic growth in less developed countries: the higher the level of concentration, the more dependent the country becomes, and the easier it will be to extract profits. Given the ‘terms of trade’ notion of the Prebisch-Singer hypothesis [54,116,117] we expect that concentrating on supplying natural resources and raw materials will still influence net profit flow negatively (see [118,119] regarding commodity dependence).

Next, we shall investigate two financial factors. High inflation will induce capital flight, hence net profit outflow. The same is expected of the number of financial crises varieties in a country: the more financial crises varieties in a country, the higher the net outflow will be [120].

The three institutional factors that we shall look into are first of all financial openness, based on the idea that more openness makes money flows easier. A negative relationship for semi-periphery and periphery is the most probable one, while it may be insignificant for core countries as in- and outflows might be balanced there. Next, one of the most important characteristics in recent decades has proved to be being a tax haven. Being a tax haven will attract profit/debt flows, hence the relationship will be positive. Lastly, investors demand strong protection of their property. Consequently, the stronger the protection of property rights, the lower the need for repatriating profits, hence the higher the net profit flow. Dreher et al. ([121]; related is [122]) showed that membership of international organizations, for instance of the International Centre for the Settlement of Investment Disputes (ICSID) of the World Bank, creates trust among investors which attracts more FDI. And when relatively more FDI is attracted, more profits will leave the country. Consequently, ICSID membership will lower the net profit flow.

The political system of a country–democracy vs. autocracy–can hardly be connected to economic growth, see [123]; none of the three arguments–security of property rights, pressure for immediate consumption, and autonomy of dictators–can be clearly linked to a political system, be it theoretically or empirically. However, a number of autocracies in poor countries have been installed by foreign interventions of countries that want to open the country for investors [124,125]. Consequently, investors will be attracted to that country, and profit outflow will be relatively large, exerting a downward pressure on net profit flow.

The final element that we shall look into is the presence of internal chaos in a country. If an–international or civil–war is fought inside a country, or anarchy exists, profits will generally be exported and inflow will be impeded, negatively influencing net profit flow.

### The model

The estimated model will be as follows:

$$\begin{array}{l} Net\;Profit\;Flow_{it}\\ \;=\beta_{0}+\beta_{1}FDIINSTOCK_{t-1}+\beta_{2}FDIOUTSTOCK_{t-1}+\beta_{3}WSP_{it}\\ \;+\beta_{4}WSP*FDIOUTSTOCK_{it-1}+\beta_{5}OPENNESS_{it}+\beta_{6}EXPCONC_{it}\\ \;+\beta_{7}RENTS_{it}+\beta_{8}FINOPEN_{it}+\beta_{9}INFLATION_{it}+\beta_{10}TAXHAVEN_{it}\\ \;+\beta_{11}ICSID_{it}+\beta_{12}AUTOCR_{it}+\beta_{13}INTCHAOS_{it}+\beta_{14}FINCRISVAR_{it-1}\\ \;+\beta_{15}1990s_{t}+\beta_{16}2000s_{t}+\varepsilon_{it} \end{array}$$ Formula 2

where:
i = country indicator;
t = year indicator;
Net Profit Flow = Net Profit Flow as percentage of GDP;
FDIINSTOCK = FDI inward stock as percentage of GDP;
FDIOUTSTOCK = FDI outward stock as percentage of GDP;
WSP = World-system Periphery (= 1);
OPENNESS = economic openness;
EXPCONC = investment concentration (Herfindahl export);
RENTS = total rents on natural resources;
FINOPEN = financial openness;
INFLATION = inflation;
TAXHAVEN = tax haven (= 1);
INTCHAOS = internal chaos;
ICSID = membership ICSID in force (= 1);
AUTOCR = level of autocracy;
*FINCRISVAR = number of financial crises varieties in a country*.

### Data

Data on the *inward and outward FDI stock* of countries are supplied by the World Bank. The data will be accepted as they are. Today, a vast body of literature exists regarding the measurement quality of FDI inward and outward stocks and flows. Two factors are mentioned: the attraction of FDI by countries and the growing importance of tax havens. Regarding the former: countries started to attract foreign investors in the 1980s by promising tax reductions, absence of restrictive regulations, subsidies etc. ([86]; [126] pp.6-8). Domestic investors, however, wanted the same perks and started what has been coined 'round-tripping'–sending the money to another country and re-importing it immediately, turning domestic investments into foreign investments in this way–with all advantages attached. The second factor are the tax havens–more precisely, secrecy jurisdictions–whose numbers grew in this very period and who made money-laundering and tax evasion easy, and also stimulated round-tripping (see [127–131]). The amount of round-tripped FDI can be substantial, the estimate for China is around 40% [17,132]. BRIC countries (Brazil, Russia, India and China) are often mentioned when it comes to high levels of round-tripping ([133]; [99], footnote 5). Of course, when FDI inflows are round-tripped the measurement of FDI stocks is also influenced, as Beugelsdijk et al. [134] and Haberly & Wojcik [135] have shown. For Austria the amount of round-tripped instock is known. It appears to be rather small: 0.2% in 2001, 0.9% in 2009 (see http://unctad.org/en/Pages/DIAE/FDI%20Statistics/FDI-Statistics-Bilateral.aspx, accessed December 2015).

World-systems group, meaning the classification into *core*, *semi-periphery and periphery*, is measured on the basis of the research of Lloyd et al. [61]. Adding the results of Mahutga [136] who investigated the period 1965–2000 is not useful as the number of missing values in the net profit flow series is very high before 1980. There have been many discussions on the notion of semi-periphery as an identifiable group of countries [54,137,138]. It consists of former core countries that lost their centrality and of former peripheral areas becoming stronger amongst others [55]. Groups 2 and 3 are coded here as belonging to the semi-periphery conforming to [61].

*Foreign investment concentration* is proxied by trade partner concentration index for exports as provided by Babones & Farabee-Siers [139], as a large part of international trade is firm-internal trade.

*Total rents on natural resources* as percentage of GDP are the sum of rents of all natural resources (coal, oil, minerals, forests etc.) a country has. They are calculated “as the difference between the price of a commodity and the average cost of producing it.” Average cost of producing includes a normal return on capital (source: http://data.worldbank.org/indicator/NY.GDP.TOTL.RT.ZS, accessed at 10-11-2016. See for the method [140] Annex 3.1 and footnote 3, p. 71).

*Openness* is measured by the sum of exports and imports of goods and services as percentage of GDP (source: World Bank).

*Financial openness*: the Chinn-Ito index (normalized) is used because of its more extensive coverage of countries and time [141,142]. *Inflation* is captured by the consumer price index as provided by the World Bank.

The data on tax havens stem from the Tax Justice Network (TJN), whose database on the Financial Secrecy Index comprises the year when a country became a tax haven (http://www.financialsecrecyindex.com/jurisdictions/database, accessed on 2-2-2016). The next variable in the finance area is the *number of financial crises varieties* (‘tally’) in a country, as composed by Reinhart & Rogoff [120].

*Security of property rights in the international economy* is signalled by membership (in force) of the ICSID of the World Bank (https://icsid.worldbank.org/en/Pages/about/Database-of-Member-States.aspx, accessed on 8-1-2016).

The political system of a country is indicated by the *autocracy* variable of the Polity IV dataset. It was decided to use the autocracy variable as it provides a more realistic measurement of the spectrum of political systems across countries. For instance, recently Gilens & Page [70] showed that the US is actually ruled by an economic elite, despite the fact that it has the highest score (10) on the democracy variable in the Polity IV dataset. There is also the mediocre score of the USA on the Perceptions of Electoral Integrity Index (https://sites.google.com/site/electoralintegrityproject4/projects/expert-survey-2; [143,144]). Concluding: the actual situation might be better described in terms of level of autocracy. Periods of foreign ‘interruption’, indicated by the code -88, were recoded to 10 (= full autocracy).

‘*Internal chaos*’ is a combination of two variables. The Correlates of War project provided the series on war periods in a country. Added are those periods that are characterised as ‘interregnum’ or ‘anarchy’, as indicated by the code -77 in the Polity IV dataset [145].

Two dummies indicating the decades of the 1990s and 2000s are added to take period effects into account and to check whether the developments in those decades have been captured; the 1980s are the reference period.

For a full account of the variables used and their sources see S2 Appendix.

### Method

The analysis will start with a description of results of the main variables, net profit flow and inward-outward FDI stock per world-system group using graphs. Countries that appeared to be outliers for their (world-system) category regarding the dependent variable and the FDI stock variables were removed. It concerns the following 6 countries: Luxembourg, Hong Kong, Switzerland, Panama and St. Kitts and Nevis; all are generally known tax havens, while Kiribati is counted as tax haven by Belgium and Portugal.

The residuals did not show the mandatory bell-shaped form as is required in parametric regression analysis. Main reason is the presence of extreme values which are either related to country characteristics–e.g. being a tax haven–or related to period–outliers can often be found in the 2000s. Removing them would create a considerable bias and reduce the value of the results. Hence, a fixed effects quantile regression was conducted. The method consists of a two-step estimation [146,147]: first a fixed-effects regression to determine the panel component. Next, the quantile regression was done with a dependent variable that is cleaned from the panel component; the quantile regression used cluster-robust standard errors to account for heteroscedasticity and autocorrelation [148] The Stata do-file can be found in S1 File. Three percentiles were estimated: 31, 43 and 85 –the means of the world-systems categories on the dependent variable corresponded with these percentiles.

The lags were based on the procedure suggested by Bellemare et al. [149].

The classification of countries into world-systems groups stems from Lloyd et al. [61]. They analysed two different years: 1980 and 2001. Hence, it is possible that countries change their position in the world-system. In the sample under investigation, for instance, China [150], Spain and the Republic Korea entered the core; they belonged to the semi-periphery before 2001. For a full account of world-system group changes see S4 Appendix. We shall focus on the constant groups to prevent influences from sample changes; results of the analyses with changing groups are available on request.

## Results and discussion

The calculations yield a series of net profit flow per country. Formally, the sum of net profit flows over the whole world should be zero. This is not the case which shows that the data are incomplete.

Table 2 gives an overview of the available data and composition of the sample.

**Table 2:** Country overview of Net Profit Flow (#).

Continent	#countries	(1) ≥ 10 observations *	(2) constant WS group **	Both (1) and (2) ***	Of which:		
NPF					Core	Semi-periphery	Periphery
Africa	54	43	41	34	0	4	30
Asia	49	25	36	20	1	9	10
Europe	49	39	40	38	7	20	11
North America	30	18	20	13	1	2	10
South America	12	11	12	11	0	7	4
Oceania	19	9	8	6	0	2	4
Total	213	145	157	122	9	44	69

# For DII see S1 Appendix Direct Investment Income Results

* Countries with 10 observations or more form the sample that will be analysed

** Countries that do not change their world-system group in 2001 (constant groups)

*** *Base sample* in the analyses (= constant groups and 10 observations or more)

68% of all countries have 10 observations or more. Oceania is least represented in the sample, while Africa, Europe and South America show a strong presence.

### Position and development per world-system group 1980–2009

**Fig 1:**
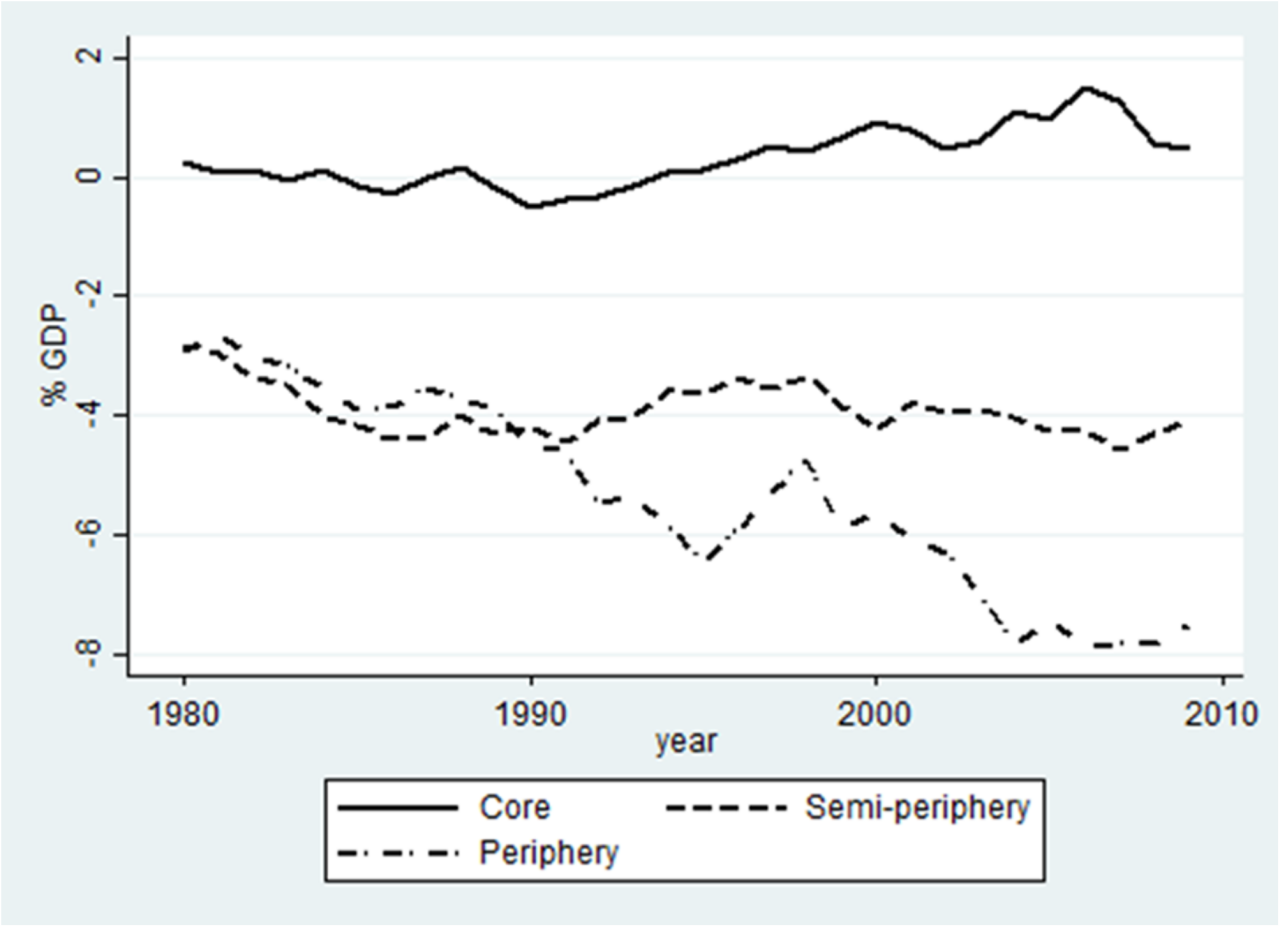
Net Profit Flow 1980–2009, base sample. Note–countries that were outliers for their world-system group (Luxembourg, Switzerland and Kiribati) were excluded. The excluded countries are generally known as tax havens; Kiribati is counted as tax haven by Portugal and Belgium.

Fig 1 pictures the position and development of the three world-system groups regarding net profit flows. All figures were constructed by calculating the unweighted average of country percentages per world-system group per year.

Fig 1 shows, first, that there is the difference between core countries on the one hand and the semi-peripheral and peripheral countries on the other. Core countries mostly show net profit inflows, while the other two groups consistently have net profit outflows. Second, positions diverged: after 1990, core countries' average net profit inflow became higher than in the previous period. Simultaneously, semi-peripheral and peripheral countries parted ways: they were on the same level in the 1980s, but the periphery's net profit outflow worsened substantially after 1990, reaching almost 8% net outflow per year after 2004. The semi-periphery stayed on a somewhat higher level of 4% net outflow.

The deterioration of the periphery position is mostly caused by debt. Terms of trade worsened in the 1980 and 1990s [151], partly due to a long-term downward development of raw materials prices on world markets [152]. It led to the Highly Indebted Poor Countries (HIPC) initiative in 1996 (http://www.imf.org/en/About/Factsheets/Sheets/2016/08/01/16/11/Debt-Relief-Under-the-Heavily-Indebted-Poor-Countries-Initiative; [81]). The debt problems also caused financial crises in the 1990s and 2000s; we shall test their influence on Net Profit Flows (NPF) in the quantile regression.

**Fig 2:**
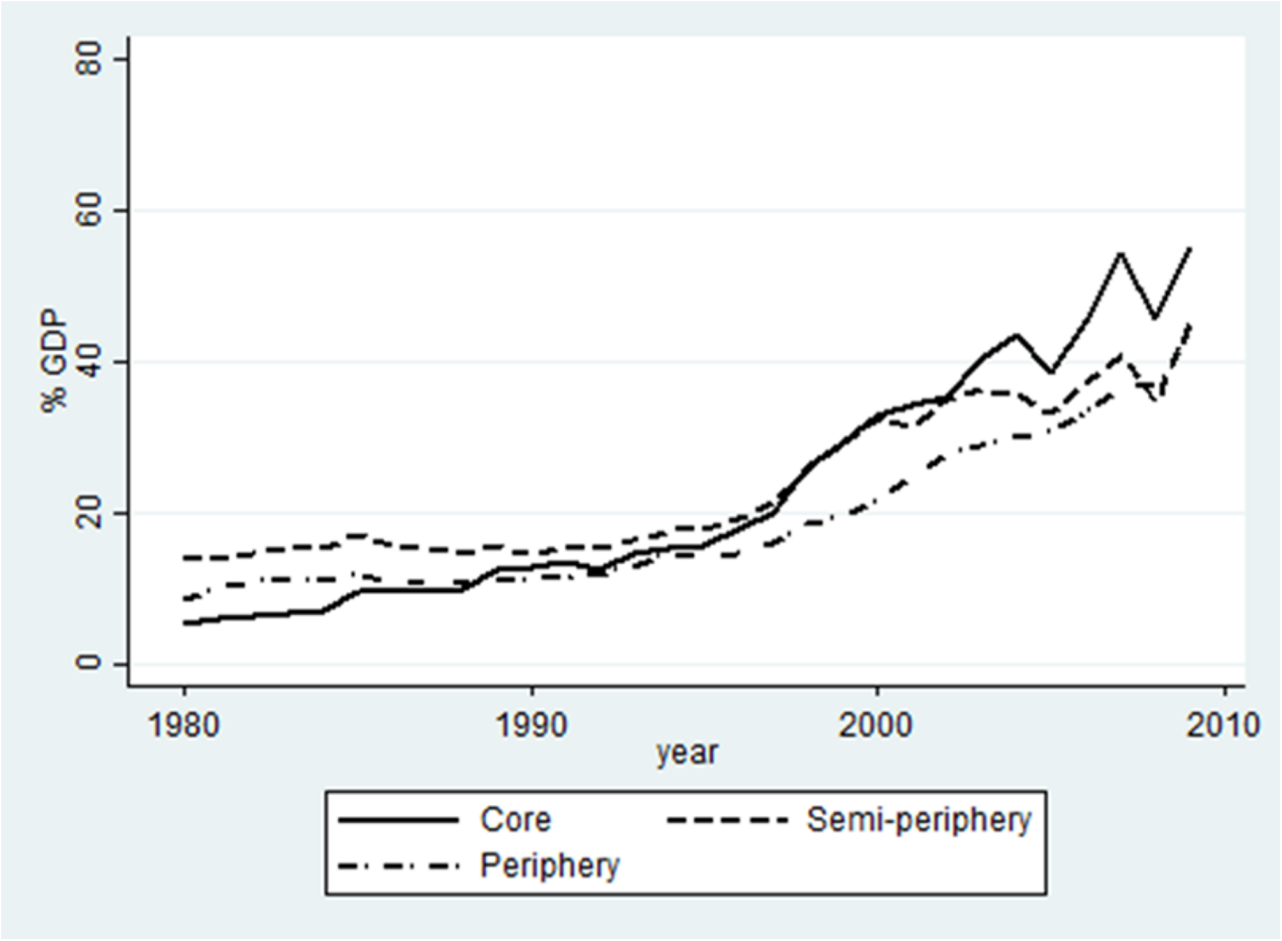
FDI inward stock 1980–2009, base sample. Note–countries that were outliers for their group (Luxembourg, Hong Kong and St. Kitts and Nevis) were excluded. All excluded countries are tax havens.

**Fig 3:**
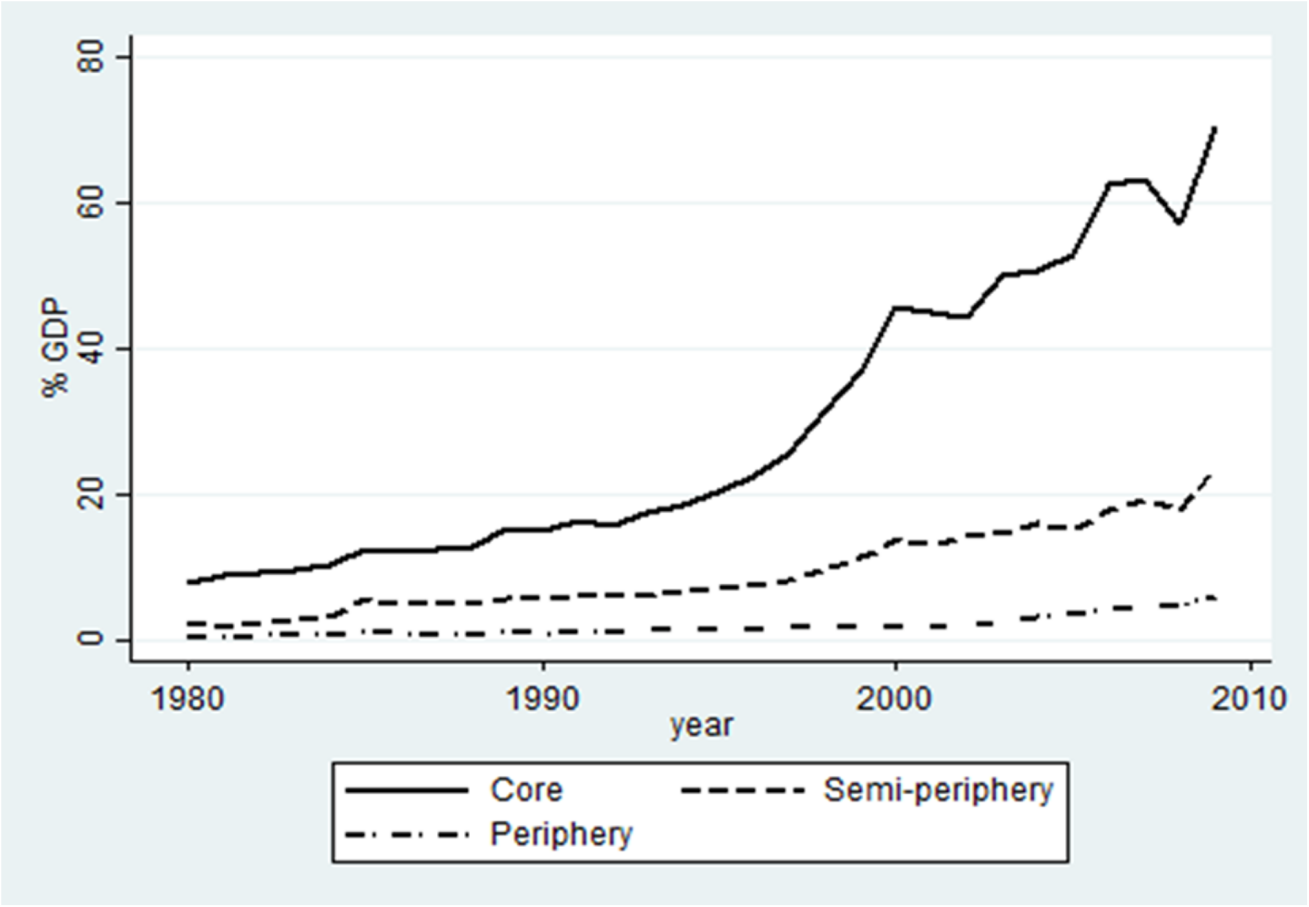
FDI outward stock 1980–2009, base sample. Note–countries that were outliers for their group (Luxembourg, Hong Kong and Panama) were excluded. All excluded countries are tax havens.

Fig 2 and Fig 3 provide a partial explanation for the developments in net profit flows (NPF). They depict the two sources of net profit flows: assuming profit repatriation, inward FDI stock leads to profit outflow and outward FDI stock creates profit inflow. Regarding inward FDI stock, we find that inward stocks have grown throughout the whole period for all world-system groups. The year 1990 is again–although somewhat less pronounced–an important year, indicating the start of a relatively fast growth of inward FDI stock. The world-system groups do not differ much regarding the levels of inward FDI stock. One of the most striking facts is the development of core countries: starting with the lowest percentage of inward FDI stock of all world-system groups, they end up having the highest level of inward stock–reflecting the fact that most FDI of developed countries has flown to other developed countries. The largest difference between the world-system groups appears to be in the level of outward FDI stock. Again, we find indications for two periods with turning point 1990; the growth of outward FDI stock is higher after 1990 in particular for the core countries, probably because of investment in Eastern European countries after the fall of the Berlin Wall [153]. Contrary to inward FDI stock, the position of the world-system groups has not changed over time: core countries show the highest level of outward FDI stock, then the semi-periphery, and lastly the peripheral countries. The growth of outward FDI stock seems to be the best explanation for the development in net profit flows.

### Panel analysis

Table 3 below shows the descriptives.

**Table 3:** Descriptives base sample #.

Variable	N	Mean	Median	S.D.	Min.	Max.
1. Net profit flow (% GDP)	2753	-4.03	-3.02	6.52	-41.52	56.52
2. FDI stock inward (% GDP)	3390	26.56	15.03	44.24	0	616.82
3. FDI stock outward (% GDP)	3403	9.59	0.98	30.08	0	535.02
4. World-system: periphery (= 1)	3467	0.56	1	0.49	0	1
5. Interaction periphery * FDI stock outward	3287	1.99	0	10.93	0	157.91
6. Openness (% GDP)	3453	79.60	69.96	46.36	6.32	432.51
7. Investment concentration (Herfindahl export)	3223	0.17	0.13	0.12	0.04	0.93
8. Total rents on natural resources (% GDP)	3549	7.45	0.53	10.97	0	78.61
9. Financial openness (1 = maximum openness)	3308	0.45	0.40	0.36	0	1
10. Inflation	3006	29.66	5.74	301.65	-13.05	11749.64
11. Tax haven (= 1)	3616	0.15	0	0.35	0	1
12. ICSID membership in force (= 1)	3619	0.69	1	0.45	0	1
13. Autocracy level Polity IV (10 = max)	3279	1.96	0	2.86	0	10
14. Number of financial crises varieties ('tally')	1643	0.98	1	1.19	0	7
15. Internal chaos (= 1)	3278	0.08	0	0.27	0	1
16. 1990s	3802	0.32	0	0.46	0	1
17. 2000s	3802	0.35	0	0.47	0	1

# Variables have not been transformed

The maximum number of observations is determined by the variable net profit flow; only the amount of observations of ‘number of financial crises varieties’ is lower, hence we shall add that variable in a separate model.

The high maximum values of inward and outward stock are related to tax havens. Economic dependencies can be very high as shown by the high maximum scores of export concentration and rents on natural resources. However, both mean and standard deviation of these variables indicate that such high dependencies are not common.

The Spearman correlations–Table 4 below–yield some interesting insights.

**Table 4:** Spearman correlations (base sample) #.

**A**											
	1	2	3	4	5	6	7	8	9	10	11
1. Net profit flow (% GDP)											
2. FDI inward stock (% GDP)	-0.26 ***										
3. FDI outward stock (% GDP)	0.13 ***	0.43 ***									
4. World-system: periphery (= 1)	-0.22 ***	-0.10 ***	-0.52 ***								
5. Periphery * FDI stock outward	-0.27 ***	0.16 ***	0.15 ***	0.63 ***							
6. Openness (% GDP)	-0.16 ***	0.51 ***	0.16 ***	0.09 ***	0.21 ***						
7. Investment concentration (Herfindahl export)	-0.24 ***	0.07***	-0.26***	0.39***	0.22***	0.18***					
8. Total rents on natural resources (% GDP)	-0.20 ***	-0.06 ***	-0.30 ***	0.15 ***	0.01	-0.26 ***	0.16 ***				
9. Financial openness (1 = max.)	0.10 ***	0.38 ***	0.50 ***	-0.29 ***	-0.06 ***	0.25 ***	-0.09 ***	-0.35 ***			
10. Inflation	-0.17 ***	-0.29 ***	-0.40 ***	0.08 ***	-0.10 ***	-0.15 ***	0.05 **	0.26 ***	-0.42 ***		
11. Tax haven (= 1)	0.14 ***	0.26 ***	0.39 ***	-0.20 ***	-0.02	0.28 ***	0.01	-0.43 ***	0.33 ***	-0.27 ***	
12. Membership ICSID in force (= 1)	-0.01	0.12 ***	0.12 ***	0.02	0.13 ***	0.08 ***	-0.13 ***	-0.04 **	0.12 ***	-0.20 ***	0.10 ***
13. Level of autocracy (10 = max)	-0.07 ***	-0.23 ***	-0.44 ***	0.42 ***	0.14 ***	-0.11 ***	0.12 ***	0.36 ***	-0.38 ***	0.08 ***	-0.29 ***
14. Number of financial crises varieties ('tally')	-0.09 ***	-0.27 ***	-0.25 ***	0.08 ***	-0.00	-0.25 ***	0.07 *	0.21 ***	-0.40 ***	0.45 ***	-0.22 ***
15. Internal chaos (= 1)	-0.11 ***	-0.19 ***	-0.16 ***	0.04 **	-0.06 **	-0.22 ***	0.02	.07 ***	-0.20 ***	0.17 ***	-0.12 ***
16. 1990s	0.05 **	-0.15 ***	-0.01	-0.00	-0.00	-0.04 **	0.01	-0.05 **	-0.01	0.07 ***	0.01
17. 2000s	-0.14 ***	0.47 ***	0.24 ***	0.00	0.14 ***	0.18 ***	-0.06 ***	0.03 *	0.21 ***	-0.27 ***	0.03*
**B**											
	12	13	14	15	16	17					
13. Level of autocracy (10 = max)	-0.05 **										
14. Number of financial crises varieties ('tally')	-0.21 ***	0.13 ***									
15. Internal chaos (= 1)	-0.11 ***	0.11 ***	0.09 ***								
16. 1990s	-0.02	-0.07 ***	0.11 ***	0.06 ***							
17. 2000s	0.16 ***	-0.18 ***	-0.25 ***	-0.10 ***	-0.50 ***						

Significance level

* ≤ .05

** ≤ .01

*** ≤ .001.

#See for base sample Table 2.

A large majority of correlations is low, showing that there is a lot of heterogeneity present in the data, and that the chance of multicollinearity is low. The correlations in general support the notions specified above: for instance, net profit flow is positively related to FDI outward stock, and negatively to FDI inward stock. The trade-related variables (rents on natural resources, openness and investment concentration) are negatively related with net profit flow; so are the number of financial crises varieties and internal chaos, but rather low. Being a tax haven is positively related. The level of autocracy and ICSID membership contradict expectations: the first one by being significant, the second one by being insignificant.

The periphery variable shows a negative correlation with net profit flow; the interaction effect of periphery*FDI outward stock is negative as well, suggesting that the effect of outward FDI stock for peripheral countries is negative. Nevertheless, the final conclusion can only be drawn after the multivariate analysis–see Table 5.

**Table 5:** Quantile regression results–base sample.

Dependent: net profit flow % GDP						
	Model 1			Model 2		
Percentiles	.31	.43	.85	.31	.43	.85
Inward FDI stock $	-.25 *** (.05)	-.28 *** (.04)	-.16 (.11)	-.33*** (.07)	-.36 *** (.04)	-.24 ** (.08)
Outward FDI stock $	.63 *** (.06)	.64 *** (.08)	.66 *** (.10)	.64 *** (.06)	.59 *** (.05)	.59 *** (.18)
Periphery (= 1)	-.41 ** (.14)	-.14 (.17)	.83 *** (.25)	-.41 (.38)	.06 (.25)	.80 * (.33)
Periphery * outward FDI stock $	-.57 *** (.05)	-.55 *** (.06)	-.81 *** (.08)	-.64 *** (.06)	-.64 *** (.05)	-.79 *** (.15)
Openness	-.30 *** (.02)	-.27 *** (.02)	-.22 *** (.03)	-.58 *** (.04)	-.55 *** (.02)	-.53 *** (.02)
Investment concentration (Herfindahl export) $	-1.53 ** (.63)	-1.94 *** (.47)	-1.15 (1.68)	-5.57 *** (.91)	-5.32 *** (.52)	-5.79 *** (.81)
Total rents on natural resources (% GDP)	-.10 *** (.00)	-.08 *** (.01)	-.06 ** (.01)	.00 (.01)	.01 (.01)	.03 *** (.01)
Financial openness (1 = max.)	-.94 *** (.28)	-.86 ** (.27)	-.14 (.41)	-1.31 ** (.44)	-1.34 *** (.34)	-.85 (.58)
Inflation	-.00 *** (.00)	-.00 *** (.00)	.00 (.00)	-.00 *** (.00)	-.00 *** (.00)	-.00 † (.00)
Tax haven (= 1)	5.76 *** (.23)	5.79 *** (.24)	5.79 *** (.35)	5.47 *** (.33)	5.58 *** (.26)	5.45 *** (.39)
Membership ICSID in force (= 1)	-.15 (.17)	-.16 (.18)	-.10 (.36)	-1.20 *** (.25)	-1.26 *** (.25)	-1.34 *** (.28)
Autocracy (10 = max.)	.06 * (.03)	.05 (.04)	.08 (.07)	.13 * (.05)	.12 (.07)	.10 ** (.03)
Internal chaos (= 1)	-.70 ** (.22)	-.56 * (.22)	-.05 (.36)	.29 (.34)	.03 (.24)	.16 (.34)
Number of financial crises varieties ('tally') $				-.28 ** (.10)	-.21 ** (.07)	.00 (.11)
1990s (= 1)	-1.12 * (.45)	-.99 * (.47)	-1.07 (.73)	.05 (.51)	-.11 (.61)	-.47 (.42)
2000s (= 1)	-1.18 ** (.38)	-.99 * (.50)	-2.15 ** (.76)	-.07 (1.16)	.31 (.67)	-.15 * (.60)
Constant	-.92 ** (.35)	-.88 * (.40)	-.11 (.56)	.54 * (.55)	.88 (.73)	1.98 *** (.32)
N obs	1875	1875	1875	1052	1052	1052
N countries	101	101	101	47	47	47
R2	.58	.58	.56	.68	.68	.66

Significance levels

† ≤ .10

* ≤ .05

** ≤ .01

*** ≤ .001

Two-step fixed effects quantile regression with cluster-robust standard errors (in parentheses). Each model has time-fixed effects (years).

$ One-year lag; lags based on [149]

The pattern of results is robust for different setups, see S5 Appendix Robustness

of significance of independent variables.

The results regarding inward and outward stock show that, as expected, profit repatriation dominates the picture, meaning that inward stock depresses net flow and outward stock has a positive influence. However, there are two exceptions. Outward stock of peripheral countries appears to contribute to capital drain instead of inflow. The total effect of outward stock for peripheral countries is close to zero or even slightly negative (adding up the coefficients of the direct and the interaction effect [154]). A test was done whether there was also a negative interaction effect for the semi-periphery. That coefficient was always positive; only the outward FDI stock of peripheral countries creates profit outflow. The second exception is inward stock of core countries: although the sign is negative (indicating outflow and repatriation), the coefficient is either insignificant or clearly lower than in the other percentiles, meaning that inward stock also partly creates capital inflow in core countries. The relatively larger standard errors support this interpretation.

At the 85^th^ percentile we also find a higher level of the interaction effect at the core level and a high positive coefficient of the periphery dummy. That is probably the result of the calculation of NPF: internal turmoil in countries like Chad and the Central African Republic probably reduces the inflow of wage remittances (or, at least, the *registered* inflow) creating a net positive profit flow as outcome.

The trade-related variables (openness, investment concentration and rents on natural resources) are negative as predicted. Their effects are lowest at the core level (85^th^ percentile), suggesting that the first two factors in particular are most harmful for periphery and semi-periphery level. The negative coefficient of ‘rents on natural resources’ shows that being a natural resources exporter is a drawback, as extensively discussed by the UNCTAD in its series on commodity dependence ([118,119]), although in the 2000s prices of natural resources went up due mainly to the strong economic growth of China (see the commodity price indices (metals, food, agricultural input) at [155]; also [17], p. 135). In the sample of model 2, however, the natural resources rents are either insignificant or have a positive effect.

Regarding the financial factors, inflation is negative as predicted, but mostly, again, for the periphery and semi-periphery. Financial crises varieties (model 2) also induce capital flight, but only for the periphery and semi-periphery, conforming to earlier research of Reinhart and Rogoff [120]. It seems a good explanation for the negative developments in the 1990s and 2000s, as the decade dummies become generally insignificant in model 2. The explanatory power of model 2 is also higher than that of model 1; however, the sample is rather small.

Institutional variables show a mixed picture. Being a tax haven exerts a consistent positive influence, and autocracy is positive several times. Financial openness indeed stimulates net profit outflow, but insignificant for the core. Membership ICSID is negative but its expected role is only significant in model 2. For internal chaos the opposite is true: relevant in the total sample–again, not for core countries–but insignificant in model 2.

The results of the analysis with DII as dependent–the robustness check, see S1 Appendix–shows that results are generally the same. The most conspicuous difference between the analysis of NPF and DII is the role tax havens play: instead of a positive coefficient, we find a negative one. Apparently, the role tax havens play seems (almost) exclusively directed at profits disguised as debt and interest payments, for instance through a ‘tax-efficient supply chain’ for profit repatriation [156], not for profit flows as such. FDI inward stock for core countries shows the same behaviour as with NPF: it is insignificant, suggesting that inward stock also generates profit inflow. Another interesting element is that the difference in coefficients between outward stock and the interaction effect is larger than with the NPF variable; the total effect of outward stock for the periphery is clearly negative.

### Discussion

Three elements warrant further discussion: the interaction effect periphery*FDI outstock (a), the periphery definition (b) and lastly the institutional element (c).

**Fig 4:**
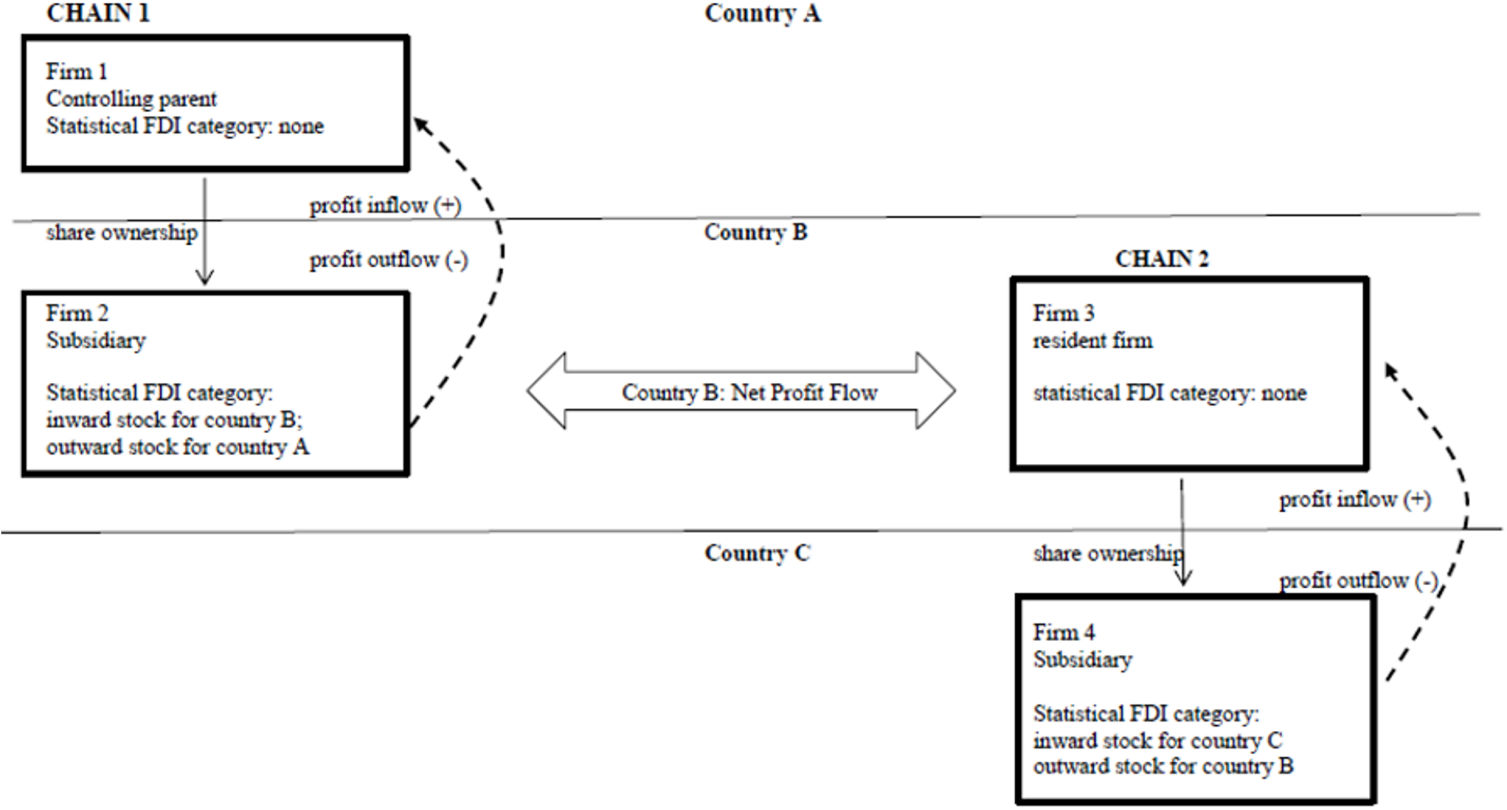
Two two-tier ownership chains matching hypotheses 1–2.

(a) The easiest way to explain the results of NPF development and the interaction effect is to picture FDI stock as stylized ownership chains. Hypotheses 1 and 2 are based on the following idea (Fig 4):

There are 4 firms; firms 2 and 4 are subsidiaries of respectively firms 1 and 3. The NPF of country B is the sum of outflow through chain 1 (firm 2) and inflow through chain 2 (firm 3).

**Fig 5:**
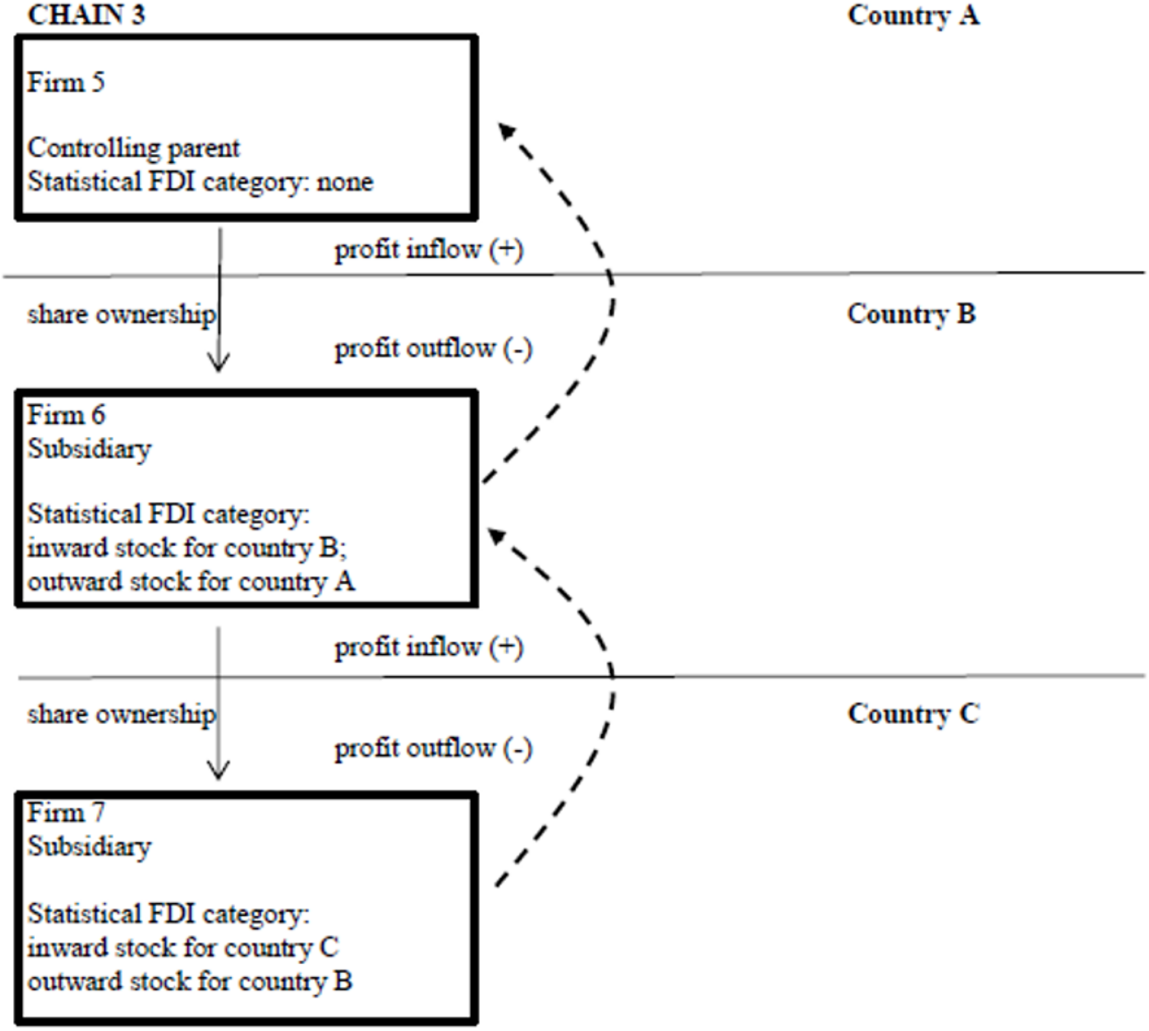
Ownership chain 3: The interaction effect in the periphery.

The interaction effect means that outstock acts as a channel for profit outflow for the periphery. In Fig 4, profit outflow through outstock might occur if firm 4 in chain 2 is located in a tax haven. However, that is probably not what the interaction effect reflects as firms in every world-system group have subsidiaries in tax havens, and the interaction effect was unique for the periphery. Fig 5 displays the chain that is probably prominent in the periphery (country B).

In this three-tier chain there is only one beneficiary owner: the top firm, firm 5. FDI outstock of firm 6 creates a profit inflow for country B, but firm 6 has to pass on that profit to the ultimate owner, firm 5. Hence, the more firms like 6 in three-tier chains in a country, the more outstock it has, and the higher the profit outflow.

**Fig 6:**
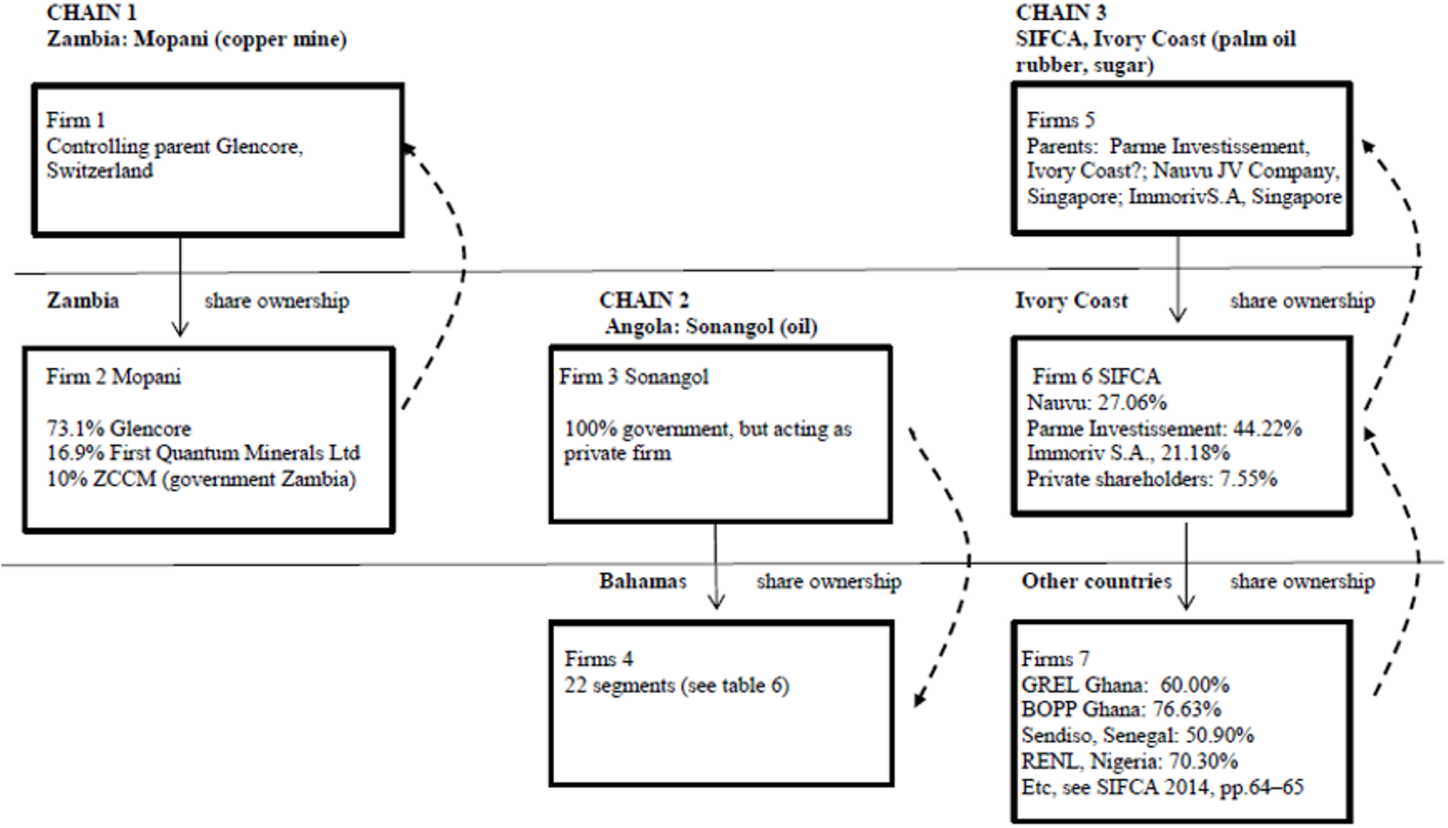
Three chains in the periphery. Sources Chain 1: http://www.glencore.com/who-we-are/about-us/our-activities-around-the-world/; [158]. Chain 2: [159], https://offshoreleaks.icij.org/#_ga=1.114935552.628152003.1481791281. Chain 3: http://olamgroup.com/news/incorporation-of-nauvu-investments-pte-ltd/#sthash.T0GsGgwt.dpbs; [160,161]. Websites accessed November-December 2016. Glencore and Immoriv S.A. are also present in the Offshore Leaks database.

Given the commodity-dependence of the periphery (69% of exports for peripheral countries ([118], p. 14); the candidate industries for such a chain are extractive industries and agriculture. The vast majority of dominant firms in the agro-food sector are core country firms, while in the extractive industries (oil and metals) dominant firms are often also in the semi-periphery ([157] p.260 ff., 289), suggesting that both core and semi-periphery exploit the periphery. Fig 6 and Table 6 provide anecdotal evidence using real-life ownership chains in the periphery (country B), all with profit outflow.

**Table 6:** Sonangol entities and operational segments.

Entity—segments	Offshore leaks database	
	Jurisdiction	Incorporation
Corporate and Financing		
Sonangol E.P.		
Sonangol Finance Ltd.		
Upstream		
Sonangol E.P.		
Sonangol Pesquisa e Producão, S.A.		
Sonangol Hidrocarbonetos Internacional, S.A.		
Sonagás–Sonangol Gás Natural, S.A.		
Midstream		
Sonangol Refinação S.A.		
Sonangol Shipping Holding, Ltd	Bahamas	24-4-2007
Sonangol Shipping Angola Ltd	Bahamas	20-7-2007
Sonangol Shipping Services Ltd	Bahamas	30-7-2007
Sonangol Chartering Services Ltd	Bahamas	30-7-2007
Sonangol LNG Shipping Service Ltd #	Bahamas	15-8-2007
Sonangol Marine Transportation Ltd	Bahamas	30-7-2007
Sonangol Marine Services Inc.		
Angola LNG Fleet Management Services LLC		
Sonangol Shipping Angola (Luanda) Limitada		
Stena Sonangol Suezmax Pool		
Sonangol Shipping Girassol Ltd	Bahamas	22-1-1999
Sonangol Huila Ltd	Bahamas	2-3-2011
Sonangol Shipping Kassanje Ltd	Bahamas	10-6-2004
Sonangol Kalandula Ltd	Bahamas	2-3-2011
Sonangol Shipping Kizomba Ltd	Bahamas	12-4-2000
Sonangol Shipping Luanda Ltd	Bahamas	12-4-2000
Sonangol Rangel Ltd	Bahamas	2-3-2011
Sonangol Porto Amboim Ltd	Bahamas	2-3-2011
Sonangol Shipping Namibe Ltd	Bahamas	16-3-2004
Sonangol Cabinda Ltd	Bahamas	2-3-2011
Sonangol Etosha Ltd	Bahamas	5-11-2009
Sonangol Benguela Ltd	Bahamas	5-11-2009
Sonangol Sambizanga Ltd	Bahamas	5-11-2009
Ngol Bengo Ltd		
Ngol Chiloango Ltd		
Ngol Zaire Ltd		
Ngol Cunene (Clyde) Ltd		
Sonangol Shipping Ngol Luena Ltd		
Sonangol Shipping Ngol Cassai Ltd		
Ngol Dande Ltd		
Ngol Kwanza Ltd		
Cumberland Ltd (Ngol Cubango)		
Downstream		
Sonagás–Sonangol Gás Natural, S.A.		
Sonangol Distribuidora, S.A.		
Sonangol Logística, Lda.		
Not in annual report as segments:		
Sonangol Suezmax Eleven Ltd	Bahamas	29-9-2015
Sonangol Suezmax Twelve Ltd	Bahamas	29-9-2015
Sonangol Cabotage Shipping Ltd	Bahamas	20-9-2007

# In the Panama Papers database it is Sonangol LNG Shipping Services Ltd, slightly different from this entry

Source: [159], p. 80 for entities and operational segments of Sonangol. Only core entities. Sonangol has other subsidiaries, see e.g. p. 85/90. Suezmax Eleven and Twelve are probably ships. The Offshore Leaks Database is https://offshoreleaks.icij.org/#_ga=1.85113689.137930281.1481842062, accessed November-December 2016. Sonangol was used as keyword, no country restrictions

Chain 1 depicts a copper mine in Zambia, controlled by mining TNC Glencore. Chain 2 shows a state-owned oil company in Angola with 22 establishments in the Bahamas; like chain 1, transfer pricing is a possibility, as are loans from the Bahama subsidiaries to the Angolan parent–the periphery then exploits the periphery here. Chain 3 (firm 6) is supposed to be characteristic for the periphery. Chain 3 is an agricultural chain: SIFCA owns subsidiaries, but is itself owned by other firms that receive profits from the total network.

(b) Earlier we defined ‘periphery’ as ‘isolates, only connected to the core’. This definition fits reality quite well as peripheral countries are indeed hardly connected to each other through trade–see S3 Appendix. Connections between peripheral countries were strengthened when they started to invest in each other [13]. However, the character of these connections will differ from those of the core. Core-core relations will probably show a mixture of chain 1 and chain 2 situations per country, but we shall hardly find chain 2 situations with profit inflow in the periphery. South-South FDI probably expanded chain 3 situations with profit outflow. We might even question whether the initiative for South-South FDI is taken by TNCs in the periphery given the fact that TNCs from core countries are generally the ultimate owner. So: is South-South investment mutual investment of peripheral countries giving opportunities for development [162], or are peripheral countries still isolates because it is just investment of core country TNCs enhancing their ‘exploitation efficiency’?

(c) Making profits all over the world requires absence of institutional barriers, be it to capital inflow, profit repatriation or foreign ownership of domestic firms [163]. The analyses of both profit variables–NPF and DII–prove the importance of institutions and policy for non-core countries in particular. The results of the NPF analysis show that financial openness is only negatively significant for the semi-periphery and the periphery: for them, financial openness creates drain of wealth. The results of the DII analysis point into the same direction. Although financial openness has a negative significant effect for all world-system groups, the core is the only group that still ends up with a net profit inflow. The core is able to ‘defend’ itself economically: there are chain 1 situations (outflow), but these are compensated for by chain 2 and 3 situations (inflow). Both semi-periphery and in particular periphery are not able to sufficiently defend themselves economically, consequently, their only defence is creating an institutional structure that corrects their weak economic position.

## Conclusion

The present paper investigated ‘net profit flow’ as a consequence of foreign investment stocks for the period 1980–2009 for three world-system groups: core, semi-periphery and periphery. Three hypotheses were tested: the presence of a hierarchy in net profit flows from high (core) to low (periphery); growing outflow in the 1990s and 2000s for both semi-periphery and periphery; and a different role for outward stock for the periphery. All three hypotheses were confirmed. Net profit inflow grew for core countries, net profit outflow for the semi-periphery and in particular the periphery. The FDI outstock of the periphery is most probably dominated by the core; it all resulted in diverging net profit flows for core on the one hand and semi-periphery and periphery on the other.

All in all, neoliberalism served core countries and TNCs well. To be precise: it served capital owners in the core well; workers in those countries have not benefited at all [164,165].[156]

## Supporting information

S1 File Stata do-file for panel quantile regression with cluster-robust standard errors. (DOCX)

S1 Appendix Direct investment income results. (DOCX)

S2 Appendix Source of data. (DOCX)

S3 Appendix Sales and purchasing dependency of core, semi-periphery and periphery 1980 and 2001. (DOCX)

S4 Appendix Change in composition of core, semi-periphery and periphery 1980–2001. (DOCX)

S5 Appendix Robustness of significance of independent variables. (DOCX)
