# Supplementary material for: Net profit flow per country from 1980 to 2009: The long-term effects of foreign direct investment
Source: PLoS One. 2017 Jun 27;12(6):e0179244. doi: 10.1371/journal.pone.0179244 (PMC5487018; doi:10.1371/journal.pone.0179244)
Supplement: S1 File — (DOCX) [file pone.0179244.s001.docx]

**S1. Stata do-file for panel quantile regression with cluster-robust standard errors**

The Stata code is:

. xtreg depvar [indepvars] [if] [in], fe [other options]

. predict helpvar, u

.drop helpvar (*optional*)

. gen newdepvar= depvar-helpvar

. qreg2 newdepvar [indepvars] [if] [in], quantile [..] cluster [*clustervar*]

.drop newdepvar (*optional*)
