# Supplementary material for: Net profit flow per country from 1980 to 2009: The long-term effects of foreign direct investment
Source: PLoS One. 2017 Jun 27;12(6):e0179244. doi: 10.1371/journal.pone.0179244 (PMC5487018; doi:10.1371/journal.pone.0179244)
Supplement: S1 Appendix — (DOCX) [file pone.0179244.s002.docx]

**S1. Table 1. Country overview of net Direct Investment Income (DII)**

| Continent | #countries | (1) ≥ 10 observations * | (2) constant WS group ** | Both (1) and (2) *** | Of which: | | |
| --- | --- | --- | --- | --- | --- | --- | --- |
| DII |  |  |  |  | Core | Semi-periphery | Periphery |
| Africa | 54 | 25 | 41 | 17 | 0 | 4 | 13 |
| Asia | 49 | 15 | 36 | 11 | 1 | 8 | 2 |
| Europe | 49 | 32 | 40 | 30 | 5 | 19 | 6 |
| North America | 30 | 11 | 20 | 8 | 1 | 1 | 6 |
| South America | 12 | 8 | 12 | 8 | 0 | 5 | 3 |
| Oceania | 19 | 6 | 8 | 5 | 0 | 2 | 3 |
| Total | 213 | 97 | 157 | 79 | 7 | 39 | 33 |

* Countries with 10 observations or more form the sample that will be analysed.

** Countries that do not change their world-system group in 2001 (constant group)

*** *Base sample* in the analysis (constant groups, 10 observations or more). Sample differs from the one of the NPF-variable.

**S1. Fig1. Net Direct Investment Income per world-system group 1980-2008, base sample**


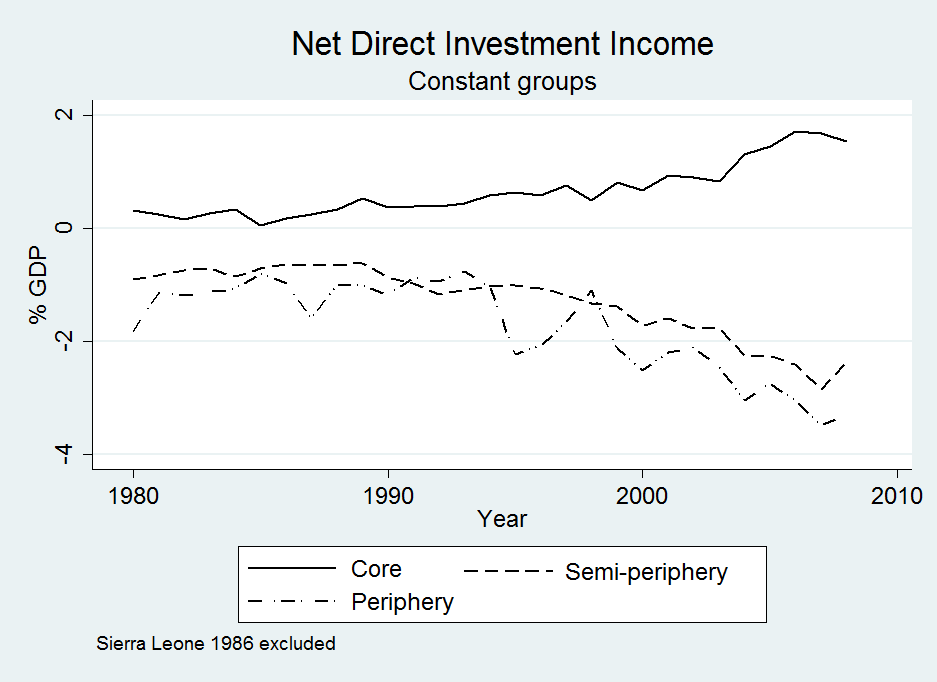


Note: Sierra Leone 1986 omitted as outlier

The DII-variable pictures the same development as the NPF variable: upwards for core countries, downwards for semi-periphery and periphery. The same hierarchy is present, although between the latter two world-system groups is smaller than for NPF. The strong downward development of the periphery is absent. Generally, the numbers are lower as DII contains only payments on intercompany debt.

Comparing the different profit variables over world-system groups yields the following zero-lag cross-correlations: core 0.84 ***, semi-periphery 0.35 †, periphery 0.81 *** (significance levels see footnote table 3 in this file). Developments for the core and periphery at the group level are about the same for both variables, while for the semi-periphery the extra debt/interest payments in the NPF variable create a different development path.

**S1. Table 2.Descriptives net DII base sample**

| Variable | N | Mean | Median | S.D. | Min. | Max. |
| --- | --- | --- | --- | --- | --- | --- |
| Direct Investment Income (% GDP) | 1679 | -1.35 | -0.59 | 2.75 | -20.92 | 8.72 |

**S1. Table 3. Quantile Regression Results net DII – base sample #**

| Dependent: net Direct Investment Income as % GDP | | | | | | |
| --- | --- | --- | --- | --- | --- | --- |
|  | Model 1 | | | Model 2 ## | | |
| Percentiles | .32 | .42 | .89 |  |  |  |
| Inward FDI stock $ | -.35 *** (.04) | -.32 *** (.02) | -.08 (.12) |  |  |  |
| Outward FDI stock $ | .52 *** (.03) | .55 *** (.04) | .66 *** (.06) |  |  |  |
| Periphery (=1) | -.04 (.07) | -.00  (.08) | .22 (.14) |  |  |  |
| Periphery * outward FDI stock $ | -.74 *** (.11) | -.86 *** (.10) | -.96 *** (.16) |  |  |  |
| Openness | -.27 *** (.01) | -.26 *** (.00) | -.21 *** (.02) |  |  |  |
| Investment concentration (Herfindahl export) $ | -0.60 ** (.23) | -.61 * (.25) | -1.30 †  (.72) |  |  |  |
| Total rents on natural resources (% GDP) | -.05 *** (.00) | -.05 *** (.00) | -.05 *** (.00) |  |  |  |
| Financial openness (1= max.) | -.84 *** (.14) | -.90 *** (.13) | -1.18 ***  (.18) |  |  |  |
| Inflation | .00 (.00) | .00 (.00) | -.00 † (.00) |  |  |  |
| Tax haven (=1) $ | -0.50 *** (.08) | -.51 *** (.07) | -.36 * (.18) |  |  |  |
| Membership ICSID in force (=1) | -.13 (.08) | -.09 (.07) | -.05 (.05) |  |  |  |
| Autocracy (10=max.) | .01 (.01) | .02 (.02) | .02 (.03) |  |  |  |
| Internal chaos (=1) | -.14 (.12) | -.09 (.18) | .54 (.36) |  |  |  |
| Number of financial crises varieties ('tally') $ |  |  |  |  |  |  |
| 1990s (=1) | 0.16 (.16) | .29 † (.15) | .01 (.39) |  |  |  |
| 2000s (=1) | .42 * (.18) | .09 (.11) | .24 (.39) |  |  |  |
| Constant | 1.48 *** (.13) | .44 *  (.17) | 1.87 *** (.45) |  |  |  |
| N obs | 1204 | 1204 | 1204 | 830 |  |  |
| N countries | 66 | 66 | 66 | 38 |  |  |
| R2 | .69 | .69 | .60 |  |  |  |

Significance levels: † ≤ .10 * ≤ .05, ** ≤ .01, *** ≤ .001

# See Table 1. The percentiles the world-system groups on the DII variable are close to the ones on NPF.

Two-step fixed effects quantile regression with cluster-robust standard errors (in parentheses). Model 1 has time-fixed effects (years). $ One-year lag; lags tested based on [130].

## Model 2 is not viable as the number of independents became larger than the number of countries.
