# Supplementary material for: Net profit flow per country from 1980 to 2009: The long-term effects of foreign direct investment
Source: PLoS One. 2017 Jun 27;12(6):e0179244. doi: 10.1371/journal.pone.0179244 (PMC5487018; doi:10.1371/journal.pone.0179244)
Supplement: S2 Appendix — (DOCX) [file pone.0179244.s003.docx]

| **S2. Table 1. Description and Source of variables** | | | |
| --- | --- | --- | --- |
| Variable | Code (if relevant) | Source | |
| * For calculation of net profit flows (1): | |  |  |
| GNP current dollars | NY.GNP.MKTP.CD | World Bank | |
| GDP current dollars | NY.GDP.MKTP.CD | World Bank | |
| Remittances received, current dollars (only in CD available) | BX.TRF.PWKR.CD.DT | World Bank | |
| Remittances paid, current dollars (only in CD available) | BM.TRF.PWKR.CD.DT | World Bank | |
| (1) For remittances Canada: <http://www5.statcan.gc.ca/cansim/a33?lang=eng&spMode=master&themeID=3764&RT=TABLE> , table 376-0101 | | | |
| World System core/semi-periphery /periphery | - | Lloyd, Mahutga & De Leeuw, J. (2009) – see the appendix | |
| FDI inward stock current $ | - | <http://knoema.com/UNCTADFDI2013/inward-and-outward-foreign-direct-investment-flows-and-stock-annual-1970-2012?action=download> | |
| FDI inward stock % | - | % GDP of host country | |
| FDI outward stock current $ | - | <http://knoema.com/UNCTADFDI2013/inward-and-outward-foreign-direct-investment-flows-and-stock-annual-1970-2012?action=download> | |
| FDI outward stock % | - | % GDP of home country | |
| Tax haven (secrecy jurisdiction) (1 = yes) | - | <http://www.financialsecrecyindex.com/jurisdictions/database> | |
| Inflation (consumer prices (annual %) | FP.CPI.TOTL.ZG | <http://databank.worldbank.org/data/home.aspx> | |
| Openness = imports + exports of goods and services as % of GDP, World Bank) | NE.EXP.GNFS.ZS,  NE.IMP.GNFS.ZS | <http://databank.worldbank.org/data/home.aspx> | |

| **S2. Table 1. Description and Source of variables (continued)** | | | | | |
| --- | --- | --- | --- | --- | --- |
| Variable | | Code (if relevant) | Source | | |
| Financial openness (Chinn-Ito) | | Kaopen normalized | Chinn & Ito, 2008  <http://web.pdx.edu/~ito/Chinn-Ito_website.htm> | | |
| Financial crises (number) | Variable 'tally' | <http://www.carmenreinhart.com/data/browse-by-topic/topics/7/> | |  |  |
| Intra-state war | - | <http://www.correlatesofwar.org/> , [Intra-StateWarData_v4.1.csv](http://www.correlatesofwar.org/COW2%20Data/WarData_NEW/Intra-StateWarData_v4.1.csv) | |  |  |
| Interregnum/anarchy | -77 in auto-/democracy variable | <http://www.systemicpeace.org/inscrdata.html> | |  |  |
| Export concentration (Herfindahl) | - | Babones, J.M. & Farabee-Siers, 2012,  <http://thedata.harvard.edu/dvn/dv/worldhistorical/faces/StudyListingPage.xhtml?mode=1&collectionId=3515> | |  |  |
| Total rents on natural resources (% GDP) | NY.GDP.TOTL.RT.ZS | <http://databank.worldbank.org/data/reports.aspx?source=2&series=NY.GDP.TOTL.RT.ZS&country>= | |  |  |
| ICSID | - | <https://icsid.worldbank.org/en/Pages/about/Member-States.aspx> | |  |  |
| Autocracy (Polity IV) | autoc | <http://www.systemicpeace.org/inscrdata.html> |  |  |  |
| Direct Investment Income, credit  (current dollars), BPM 5 | BXIID | [http://elibrary-data.imf.org](http://elibrary-data.imf.org/) |  |  |  |
| Direct Investment Income, debit  (current dollars), BPM 5 | BMIID | [http://elibrary-data.imf.org](http://elibrary-data.imf.org/) |  |  |  |
