# Supplementary material for: Net profit flow per country from 1980 to 2009: The long-term effects of foreign direct investment
Source: PLoS One. 2017 Jun 27;12(6):e0179244. doi: 10.1371/journal.pone.0179244 (PMC5487018; doi:10.1371/journal.pone.0179244)
Supplement: S3 Appendix — (DOCX) [file pone.0179244.s004.docx]

**S3. Table. Sales and purchasing dependency of core, semi-periphery and periphery, 1980 and 2001, in percentages (1)**

| S3. Table 1. Sales Dependency Core – Semi-periphery – Periphery, 1980 | | | | | |
| --- | --- | --- | --- | --- | --- |
|  | Core | Semi-periphery | Periphery |  |  |
| Core | 82.2 | 16.5 | 1.3 | 100 |  |
| Semi-periphery | 85.4 | 13.6 | 1.0 | 100 |  |
| Periphery | 88.1 | 10.1 | 1.8 | 100 |  |
|  |  |  |  |  |  |

| S3. Table 2. Sales Dependency Core – Semi-periphery – Periphery, 2001 | | | | | |
| --- | --- | --- | --- | --- | --- |
|  | Core | Semi-periphery | Periphery |  |  |
| Core | 85.0 | 13.8 | 1.2 | 100 |  |
| Semi-periphery | 75.4 | 22.2 | 2.4 | 100 |  |
| Periphery | 75.1 | 20.4 | 4.5 | 100 |  |
|  |  |  |  |  |  |

| S3. Table 3. Purchasing Dependency Core – Semi-periphery – Periphery, 1980 | | | | | |
| --- | --- | --- | --- | --- | --- |
|  | Core | Semi-periphery | Periphery |  |  |
| Core | 80.8 | 84.4 | 83.8 |  |  |
| Semi-periphery | 18.2 | 15.0 | 14.9 |  |  |
| Periphery | 1.0 | 0.6 | 1.3 |  |  |
|  | 100 | 100 | 100 |  |  |

| S3. Table 4. Purchasing Dependency Core – Semi-periphery – Periphery, 2001 | | | | | |
| --- | --- | --- | --- | --- | --- |
|  | Core | Semi-periphery | Periphery |  |  |
| Core | 83.5 | 73.7 | 67.7 |  |  |
| Semi-periphery | 15.8 | 25.2 | 29.6 |  |  |
| Periphery | 0.7 | 1.1 | 2.7 |  |  |
|  | 100 | 100 | 100 |  |  |

(1) All calculations based on tables 3 and 4 in Lloyd et al., 2009.

The tables show the sales and purchasing dependency of the core, semi-peripheral and peripheral countries. The core has become more dependent upon itself, as the rising sales and purchasing dependencies show. On the other hand, the sales dependency of semi-periphery and periphery towards each other grew, and their sales dependency of the core went down. The same goes for the purchasing dependency of both country groups.

This development seems a little anomalous, as many core countries have outsourced production. A part of the anomaly can be explained by the change of position in the world system, and by China in particular: this country entered the core in 2001 and left the semi-periphery.
