# Supplementary material for: Net profit flow per country from 1980 to 2009: The long-term effects of foreign direct investment
Source: PLoS One. 2017 Jun 27;12(6):e0179244. doi: 10.1371/journal.pone.0179244 (PMC5487018; doi:10.1371/journal.pone.0179244)
Supplement: S4 Appendix — (DOCX) [file pone.0179244.s005.docx]

**S4. Table 1. Change in composition of core, semi-periphery and periphery 1980-2001**

| Country | 1980 | 2001 |
| --- | --- | --- |
| Afghanistan | Periphery | Periphery |
| Albania | Periphery | Periphery |
| Algeria | Semi-periphery | Semi-periphery |
| American Samoa | - | - |
| Andorra | - | - |
| Angola | Periphery | Periphery |
| Antigua and Barbuda | - | - |
| Argentina | Semi-periphery | Semi-periphery |
| Armenia | Semi-periphery | Periphery |
| Aruba | - | - |
| Australia | Semi-periphery | Semi-periphery |
| Austria | Semi-periphery | Semi-periphery |
| Azerbaijan | - | Periphery |
| Bahamas, The | Semi-periphery | Periphery |
| Bahrain | Semi-periphery | Periphery |
| Bangladesh | Periphery | Periphery |
| Barbados | Periphery | Periphery |
| Belarus | - | Semi-periphery |
| Belgium | Core | Core |
| Belize | Periphery | Periphery |
| Benin | Periphery | Periphery |
| Bermuda | Periphery | Periphery |
| Bhutan | Periphery | Periphery |
| Bolivia | Periphery | Periphery |
| Bosnia and Herzegovina | - | Periphery |
| Botswana | - | - |
| Brazil | Semi-periphery | Semi-periphery |
| Brunei Darussalam | Periphery | Periphery |
| Bulgaria | Semi-periphery | Semi-periphery |
| Burkina Faso | Periphery | Periphery |
| Burundi | Periphery | Periphery |
| Cambodia | Periphery | Periphery |
| Cameroon | Periphery | Periphery |
| Canada | Semi-periphery | Semi-periphery |
| Cape Verde | - | - |
| Cayman Islands | Periphery | Periphery |
| Central African Republic | Periphery | Periphery |
| Chad | Periphery | Periphery |
| Channel Islands | - | - |
| Chile | Semi-periphery | Semi-periphery |
| China | Semi-periphery | Core |
| Colombia | Semi-periphery | Semi-periphery |
| Comoros | Periphery | Periphery |
| Congo, Dem. Rep. | Periphery | Periphery |
| Congo, Rep. | Periphery | Periphery |
| Costa Rica | Periphery | Periphery |

**S4. Table 1. Change in composition of core, semi-periphery and periphery 1980-2001 (continued)**

| Country | 1980 | 2001 |
| --- | --- | --- |
| Cote d'Ivoire | Semi-periphery | Periphery |
| Croatia | - | Semi-periphery |
| Cuba |  | Periphery |
| Cyprus | Periphery | Periphery |
| Czech Republic | Semi-periphery | Semi-periphery |
| Denmark | Semi-periphery | Semi-periphery |
| Djibouti | Periphery | Periphery |
| Dominica | - | - |
| Dominican Republic | Periphery | Periphery |
| Ecuador | Semi-periphery | Semi-periphery |
| Egypt, Arab Rep. | Semi-periphery | Periphery |
| El Salvador | Periphery | Periphery |
| Equatorial Guinea | Periphery | Semi-periphery |
| Eritrea | - | - |
| Estonia | - | Periphery |
| Ethiopia | Periphery | Periphery |
| Faeroe Islands | - | - |
| Fiji | Periphery | Periphery |
| Finland | Periphery | Periphery |
| France | Core | Core |
| French Polynesia | - | - |
| Gabon | Periphery | Periphery |
| Gambia, The | Periphery | Periphery |
| Georgia | - | Periphery |
| Germany | Core | Core |
| Ghana | Periphery | Periphery |
| Gibraltar | Periphery | Periphery |
| Greece | Semi-periphery | Semi-periphery |
| Greenland | Periphery | Periphery |
| Grenada | - | - |
| Guam | - | - |
| Guatemala | Periphery | Periphery |
| Guinea | Periphery | Periphery |
| Guinea-Bissau | Periphery | Periphery |
| Guyana | Periphery | Periphery |
| Haiti | Periphery | Periphery |
| Honduras | Periphery | Periphery |
| Hong Kong SAR, China | Semi-periphery | Semi-periphery |
| Hungary | Semi-periphery | Semi-periphery |
| Iceland | Periphery | Periphery |
| India | Semi-periphery | Semi-periphery |
| Indonesia | Semi-periphery | Semi-periphery |
| Iran, Islamic Rep. | Semi-periphery | Semi-periphery |
| Iraq | Semi-periphery | Periphery |
| Ireland | Semi-periphery | Semi-periphery |

**S4. Table 1. Change in composition of core, semi-periphery and periphery 1980-2001 (continued)**

| Country | 1980 | 2001 |
| --- | --- | --- |
| Isle of Man | - | - |
| Israel | Semi-periphery | Semi-periphery |
| Italy | Core | Core |
| Jamaica | Periphery | Periphery |
| Japan | Core | Core |
| Jordan | Periphery | Periphery |
| Kazakhstan | - | Semi-periphery |
| Kenya | Semi-periphery | Semi-periphery |
| Kiribati | Periphery | Periphery |
| Korea, Dem. Rep. | Periphery | Periphery |
| Korea, Rep. | Semi-periphery | Core |
| Kosovo | - | - |
| Kuwait | Semi-periphery | Semi-periphery |
| Kyrgyz Republic | - | Periphery |
| Lao PDR | Periphery | Periphery |
| Latvia | - | Periphery |
| Lebanon | Periphery | Periphery |
| Lesotho | - | - |
| Liberia | Periphery | Periphery |
| Libya | Semi-periphery | Semi-periphery |
| Liechtenstein | - | - |
| Lithuania | - | Semi-periphery |
| Luxembourg | Core | Core |
| Macao SAR, China | - | Periphery |
| Macedonia, FYR | - | Periphery |
| Madagascar | Periphery | Periphery |
| Malawi | Periphery | Periphery |
| Malaysia | Semi-periphery | Semi-periphery |
| Maldives | Periphery | Periphery |
| Mali | Periphery | Periphery |
| Malta | Periphery | Periphery |
| Marshall Islands | - | - |
| Mauritania | Periphery | Periphery |
| Mauritius | Periphery | Periphery |
| Mayotte | - | - |
| Mexico | Semi-periphery | Semi-periphery |
| Micronesia, Fed. Sts. | - | - |
| Moldova | - | Periphery |
| Monaco | - | - |
| Mongolia | Periphery | Periphery |
| Montenegro | - | - |
| Morocco | Periphery | Semi-periphery |
| Mozambique | Periphery | Periphery |
| Myanmar | Periphery | Periphery |
| Namibia | - | - |

**S4. Table 1. Change in composition of core, semi-periphery and periphery 1980-2001 (continued)**

| Country | 1980 | 2001 |
| --- | --- | --- |
| Nepal | Periphery | Periphery |
| Netherlands | Core | Core |
| Netherlands Antilles |  |  |
| New Caledonia | Periphery | Periphery |
| New Zealand |  |  |
| Nicaragua | Periphery | Periphery |
| Niger | Periphery | Periphery |
| Nigeria | Semi-periphery | Semi-periphery |
| Northern Mariana Islands | - | - |
| Norway | Semi-periphery | Semi-periphery |
| Oman | Periphery | Semi-periphery |
| Pakistan | Semi-periphery | Semi-periphery |
| Palau | - | - |
| Panama | Periphery | Periphery |
| Papua New Guinea | Periphery | Periphery |
| Paraguay | Periphery | Periphery |
| Peru | Semi-periphery | Semi-periphery |
| Philippines | Semi-periphery | Semi-periphery |
| Poland | Semi-periphery | Semi-periphery |
| Portugal | Semi-periphery | Semi-periphery |
| Puerto Rico | - | - |
| Qatar | Semi-periphery | Periphery |
| Romania | Semi-periphery | Semi-periphery |
| Russian Federation | Semi-periphery | Semi-periphery |
| Rwanda | Periphery | Periphery |
| Samoa | - | - |
| San Marino | - | - |
| Sao Tome and Principe | - | - |
| Saudi Arabia | Semi-periphery | Semi-periphery |
| Senegal | Periphery | Periphery |
| Serbia | - | - |
| Seychelles | Periphery | Periphery |
| Sierra Leone | Periphery | Periphery |
| Singapore | Semi-periphery | Core |
| Slovak Republic | Semi-periphery | Semi-periphery |
| Slovenia | - | Semi-periphery |
| Solomon Islands | Periphery | Periphery |
| Somalia | Periphery | Periphery |
| South Africa | Semi-periphery | Semi-periphery |
| Spain | Semi-periphery | Core |
| Sri Lanka | Periphery | Periphery |
| St. Kitts and Nevis | Periphery | Periphery |
| St. Lucia | - | - |
| St. Vincent and the Grenadines | - | - |

**S4. Table 1. Change in composition of core, semi-periphery and periphery 1980-2001 (continued)**

| Country | 1980 | 2001 |
| --- | --- | --- |
| Sudan | Periphery | Periphery |
| Suriname | Periphery | Periphery |
| Swaziland | - | - |
| Sweden | Semi-periphery | Semi-periphery |
| Switzerland | Semi-periphery | Semi-periphery |
| Syrian Arab Republic | Semi-periphery | Periphery |
| Tajikistan | - | Periphery |
| Tanzania | Periphery | Periphery |
| Thailand | Semi-periphery | Semi-periphery |
| Timor-Leste | - | - |
| Togo | Periphery | Periphery |
| Tonga | - | - |
| Trinidad and Tobago | Semi-periphery | Semi-periphery |
| Tunisia | Semi-periphery | Semi-periphery |
| Turkey | Semi-periphery | Semi-periphery |
| Turkmenistan | - | Periphery |
| Turks and Caicos Islands | Periphery | Periphery |
| Tuvalu | - | - |
| Uganda | Periphery | Periphery |
| Ukraine | - | Semi-periphery |
| United Arab Emirates | Semi-periphery | Semi-periphery |
| United Kingdom | core | Core |
| United States | core | Core |
| Uruguay | Periphery | Periphery |
| Uzbekistan | - | Periphery |
| Vanuatu | - | - |
| Venezuela, RB | Semi-periphery | Semi-periphery |
| Vietnam | Periphery | Semi-periphery |
| Virgin Islands (U.S.) | - | - |
| West Bank and Gaza | - | - |
| Yemen, Rep. | Periphery | Periphery |
| Zambia | Periphery | Periphery |
| Zimbabwe | Periphery | Periphery |

Classification based on Lloyd et al., 2009, appendix. Core = group 1, semi-periphery = groups 2 and 3, periphery = groups 4, 5 and 6.

- = not incorporated in the network analysis. Eastern European countries might not have existed in 1980.
