# Supplementary material for: Net profit flow per country from 1980 to 2009: The long-term effects of foreign direct investment
Source: PLoS One. 2017 Jun 27;12(6):e0179244. doi: 10.1371/journal.pone.0179244 (PMC5487018; doi:10.1371/journal.pone.0179244)
Supplement: S5 Appendix — (DOCX) [file pone.0179244.s006.docx]

**S5. Table 1. Robustness of significance of independent variables**

|  | Independent | Correlation | Model # & | | | | | | | | | | |  |  |  |  | | |
| --- | --- | --- | --- | --- | --- | --- | --- | --- | --- | --- | --- | --- | --- | --- | --- | --- | --- | --- | --- |
|  |  |  | i | ii | iii | iv | v | vi | vii | viii | ix | x | xi | xii | xiii (c) | xiv (c) | |  |  |
| 1 | Periphery * FDI outward stock (a) $ | -0.27 *** | A | A | A | A | A | A | A | A | A | A | A | A | A | A | |  |  |
| 2 | World system: periphery (=1) (a) | -0.22 *** | 31-85 | 85 | 31-85 | 85 | 31-85 | 31-85 | 31-85 | 31-85 | 31-85 | 31-85 | 31-85 | 31-85 | 31-85 | 85 | |  |  |
| 3 | FDI outward stock (a) $ | 0.13 *** | A | A | A | A | A | A | A | A | A | A | A | A | A | A | |  |  |
| 4 | FDI inward stock $ | -0.26 *** |  | A | A | A | A | A | A | A | 31-43 | 31-43 | 31-43 | 31-43 | 31-43 | A | |  |  |
| 5 | Investment concentration (Herfindahl export) $ | -0.24 *** |  |  | - | - | - | - | - | - | 31-43 | 31-43 | 31-43 | 31-43 | 31-43 | A | |  |  |
| 6 | Total rents on natural resources | -0.20 *** |  |  |  | A | A | A | A | A | A | A | A | A | A | 85 | | |  |
| 7 | Inflation | -0.17 *** |  |  |  |  | 31-43 | 31-43 | A | A | 31-43 | 31-43 | 31-43 | 31-43 | 31-43 | A | | |  |
| 8 | Openness | -0.16 *** |  |  |  |  |  | A | A | A | A | A | A | A | A | A | | |  |
| 9 | Tax haven | 0.14 *** |  |  |  |  |  |  | A | A | A | A | A | A | A | A | | |  |
| 10 | 2000s | -0.14 *** |  |  |  |  |  |  |  | A | A | A | A | A | A | 85 | | |  |
| 11 | Internal chaos (=1) | -0.11 *** |  |  |  |  |  |  |  |  | 31-43 | 31-43 | 31-43 | 31-43 | 31-43 | - | | |  |
| 12 | Financial openness (1=max) | 0.10 *** |  |  |  |  |  |  |  |  |  | 31-43 | 31-43 | 31-43 | 31-43 | 31-43 | | |  |
| 13 | Level of autocracy (10=max) | -0.07 *** |  |  |  |  |  |  |  |  |  |  | 31 | 31 | 31 | 31-85 | | |  |
| 14 | 1990s | 0.05 ** |  |  |  |  |  |  |  |  |  |  |  | A | 31-43 | - | | |  |
| 15 | Membership ICSID in force (=1) | -0.01 |  |  |  |  |  |  |  |  |  |  |  |  | - | A | | |  |
|  |  |  |  |  |  |  |  |  |  |  |  |  |  |  |  |  | | |  |
| 16 | Number of financial crises varieties (‘tally’) (b) | -0.09 *** |  |  |  |  |  |  |  |  |  |  |  |  |  | 31-43 | | |  |
|  | N of observations |  | 2489 | 2446 | 2414 | 2414 | 2155 | 2120 | 1908 | 1908 | 1901 | 1875 | 1875 | 1875 | 1875 | 1052 | | |  |
|  | N of countries |  | 119 | 119 | 118 | 118 | 111 | 111 | 104 | 104 | 101 | 101 | 101 | 101 | 101 | 47 | | |  |

# The values of the dependent variable (Net Profit Flows as % GDP) differ per model given the method (see Support file 2, Stata do file). Cluster-robust standard errors, year-fixed effects.

& The cells indicate for which percentile the variables were significant at the level of ≤ .10. A = all; - = none.

(a) These three variables were jointly added.

(b) This variable was again added as last one, given its influence on the number of observations.

(c) Models as in the main text.

The table above shows the significance of the independent variables in different setups. The models were composed by adding independents to the equation depending on the height of the correlation with the dependent: highest first, lowest last. There are two deviations from this rule: the first three variables were jointly added as they belong together because of the interaction effect, and the ‘tally’ variable was added last due to its effect on the number of observations.

Generally, the variables show a consistent pattern in the different setups – 2000s, export concentration and FDI inward stock less so. FDI outward stock, the interaction effect, rents on natural resources, tax haven and openness are effective in all country groups, while export concentration, financial openness, inflation and internal chaos influence the net profit flows in peripheral and semi-peripheral countries, but not in core countries. The same goes for financial crises: they hurt those two country groups, and show no significance for net profit flows in core countries.
